# Single-enzyme redox-neutral oxidation of alcohols to carboxylic acids using alcohol dehydrogenases

**DOI:** 10.1039/d5cy01223f

**Published:** 2025-12-10

**Authors:** Matteo Damian, Zheng Wei, Vasilis Tseliou, Francesco G. Mutti

**Affiliations:** a Van't Hoff Institute for Molecular Sciences, HIMS-Biocat, University of Amsterdam Science Park 904 1098 XH The Netherlands f.mutti@uva.nl

## Abstract

Oxidation of primary alcohols to carboxylic acids is a fundamental reaction in organic chemistry, traditionally dependent on toxic oxidants and often limited by poor selectivity. In this study, we demonstrate the multifunctional capability of some alcohol dehydrogenases (ADHs) to catalyze both alcohol and aldehyde oxidation while regenerating their NAD^+^ cofactor through concomitant reduction of acetone. Screening of a panel of ADHs revealed that the enzymes from *Paracoccus pantotrophus* (*Pp*-ADH) and *Aromatoleum aromaticum* (*Aa*-ADH) have strong overoxidation activity to carboxylic acids. The biocatalytic method was assessed for the efficient oxidation of a panel of 27 structurally diverse primary alcohols into carboxylic acids using a single enzyme, with minimal workup and without the need for further purification. The biotransformation was also scaled up using cell-free extracts, while maintaining high yields. *In silico* studies provided insights into substrate tolerance, highlighting the structural features that govern enzyme activity. This biocatalytic method provides a scalable, selective, and environmentally friendly alternative to conventional oxidation strategies for primary alcohols to carboxylic acids.

## Abstract 2

This work uncovers alcohol dehydrogenases that enable green, efficient, and selective single-enzyme oxidation of alcohols to carboxylic acids using acetone.

## Graphical abstract

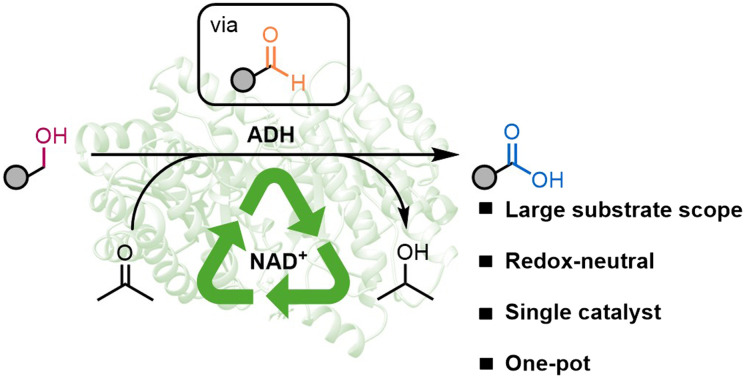

## Introduction

Carboxylic acids have important biological functions and, therefore, have relevant applications in medicine, food, and cosmetics.^1–3^ They also find applications as insecticides, dye intermediates, coatings, plasticizers, monomers and solvents.^4^ Carboxylic acids have crucial importance as intermediates in various areas of catalysis.^5–7^ Traditionally, the transformation of alcohols into carboxylic acids relies on stoichiometric amounts of environmentally unfriendly oxidants,^8^ such as permanganate,^9^ Cr(vi) reagents,^10^ pyridinium dichromate,^11^ ruthenium tetraoxide and other ruthenium compounds,^12^ chlorite or hypochlorite,^8^ and TEMPO-mediated oxidations.^13^ Other examples in the literature have shown the possibility of producing carboxylic acids from aldehydes using metal catalysts and air as the oxidant.^13–15^ One other limitation of these chemical methods is the imperfect selectivity when different oxidizable functional groups are present within the same molecule. Aiming at improving the greenness and the selectivity of the catalytic oxidation of primary alcohols to carboxylic acids, alternative approaches based on transition metals, organocatalysts, nanocatalysts, and electrochemical methods have been developed.^4^ However, these approaches frequently suffer from other practical limitations, including the requirement for high-boiling solvents, elevated catalyst loadings, or scalability issues.

**Scheme 1:**
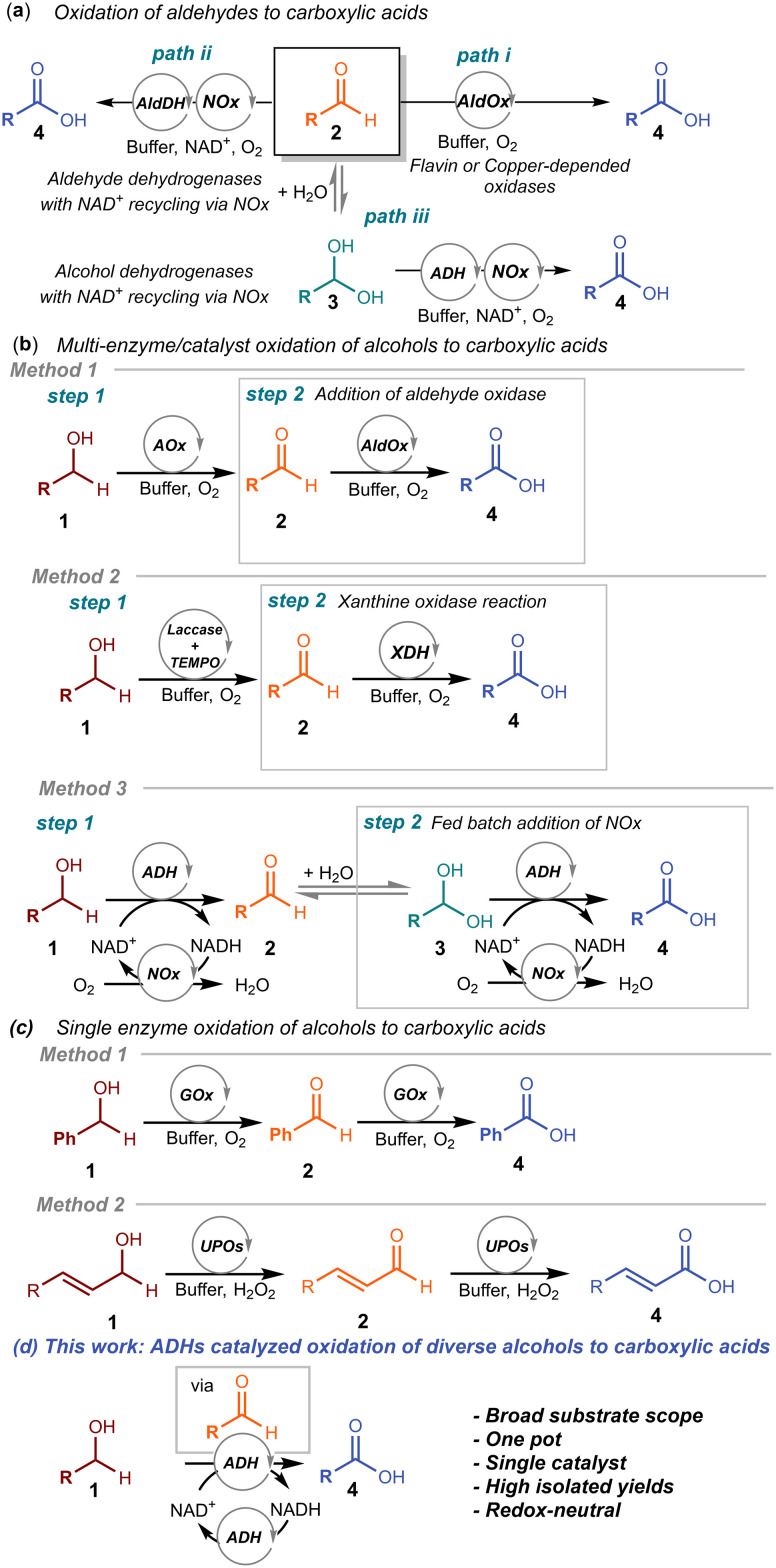
Reported methodologies for the biocatalytic synthesis of carboxylic acids. a) Oxidation of aldehydes using metal-dependent or flavin-dependent aldehyde oxidases (AldOx), or aldehyde dehydrogenases (AldDH), or alcohol dehydrogenases (ADH). b) Oxidation of alcohols to carboxylic acids using alcohol oxidase (AOx) and aldehyde oxidase (AldOx, method 1), laccases with TEMPO as mediator and xanthine dehydrogenase (XDH, method 2), and alcohol dehydrogenase (ADH) mediated overoxidation of alcohols to carboxylic acids using a fed-batch addition of a nicotinamide adenine dinucleotide oxidase (NOx, method 3). c) Oxidation of benzylic alcohols to benzoic acids catalyzed by a galactose oxidase variant (GOx, method 1), and oxidation of cinnamic alcohols to cinnamic acids catalyzed by unspecific peroxygenases (UPO). d) This work: a one-enzyme redox-neutral system for the oxidation of alcohols to carboxylic acids using acetone as a sacrificial cosubstrate to recycle the NAD^+^ cofactor.

Biocatalysis offers a promising alternative thanks to its high selectivity, mild reaction conditions, and compatibility with green chemistry principles.^16^ In this context, enzymatic oxidation of aldehydes to carboxylic acids has been demonstrated using flavin- or metal-dependent oxidases (Scheme 1a, path i)^17,18^ or whole cells of *Comamonas testosteroni* SC1588.^19^ Unfortunately, these systems are typically substrate-specific for 5-hydroxymethylfurfural, limiting their application in organic synthesis. A more general approach was achieved using aldehyde dehydrogenases (AldDHs) coupled with a nicotinamide adenine dinucleotide (NADH) oxidase (NOx) for NAD^+^ regeneration (Scheme 1a, path ii), as reported by our group and others in the last decade.^20–28^ Recently, Han *et al.* reported a whole-cell approach using the recently discovered succinic semialdehyde dehydrogenase from *Klebsiella pneumoniae* (*Kp*SSADH) for converting various heteroatom-containing cyclic aldehydes into the corresponding carboxylic acids without the need for any additional enzyme for recycling the cofactor.^29^ Additionally, alcohol dehydrogenases (ADHs) have been combined with NADH oxidase (NOx) for oxidative dynamic kinetic resolution of profens,^30^ and later for the oxidation of aldehydes to carboxylic acids *via* the hydrate of the aldehyde intermediate (Scheme 1a, path iii).^31,32^

While these processes efficiently oxidize aldehydes, the direct oxidation of alcohols to carboxylic acids remains a challenge. One approach employed a tandem system combining an engineered *Fusarium graminearum* galactose oxidase (GOase) with the aldehyde oxidase PaoABC, while requiring a catalase enzyme for H_2_O_2_ removal (Scheme 1b, method 1).^33^ This multi-enzymatic system suffered from low performance at high alcohol concentrations, requiring a large amount of catalyst loading. As an alternative (Scheme 1b, method 2), the oxidation of alcohols to aldehydes, catalyzed by laccases in combination with TEMPO as a mediator,^34^ was combined with the further oxidation of aldehydes to carboxylic acids using a xanthine dehydrogenase (XDH).^35^ In another study, an alcohol dehydrogenase (ADH) from *Halomonas elongata* was found to be capable of catalyzing both alcohol and aldehyde oxidation. To achieve full oxidation to carboxylic acids, the enzyme was used in tandem with NADH oxidase (NOx). However, despite the dual activity of the ADH, the use of a second enzyme remained necessary, and the low stability of NOx required a fed-batch addition strategy to sustain activity throughout the reaction (Scheme 1b, method 3).^36^

Another interesting approach involves the use of an alcohol oxidase (AOx), in particular galactose oxidase (GOx), which yielded carboxylic acids starting directly from the alcohol, with a single enzyme (Scheme 1c, method 1). However, this approach is basically limited to benzyl alcohols.^37,38^ Notably, all these methods based on oxidases or NOx for cofactor recycling require only dioxygen from air as environmentally benign oxidant. When an H_2_O_2_-forming NOx is employed, the addition of catalase is necessary to prevent enzyme deactivation. In our group, we normally use water-forming NOx, such as the enzyme described in Higuchi *et al.*^39^

Similar to oxidases, unspecific peroxygenases (UPOs) have been employed for the oxidation of alcohols to carboxylic acids, but their application is likewise restricted by a narrow substrate scope (Scheme 1c, method 2).^40–42^

In this study, we harness the multiple functionality of alcohol dehydrogenases (ADHs) to catalyze both the oxidation of alcohol to aldehyde and the subsequent further oxidation of aldehyde to carboxylic acid, while simultaneously combining the reduction of acetone, as a sacrificial cosubstrate, to isopropanol for NAD^+^ cofactor regeneration (Scheme 1d). This single-enzyme redox-neutral system eliminates the need for an additional enzyme for cofactor recycling, thereby simplifying the process, improving the overall efficiency of the process, and reducing costs. Although the use of isopropanol or acetone as sacrificial cosubstrates for cofactor recycling in ADH-catalyzed carbonyl reduction and alcohol oxidation is both established and advantageous, only a subset of ADHs can accept these cosubstrates or remain stable in their presence.^43–45^

## Results and discussion

To establish the biocatalytic oxidation system, we initially tested hexanol (1b, 5 mM) as a model substrate in the presence of NOx (10 μM) and NAD^+^ (0.5 mM), together with a panel of ten ADHs (10 μM). Among these, four enzymes namely from *Pichia finlandica* (*Pf*-ADH),^46,47^*Paracoccus pantotrophus* (*Pp*-ADH),^48,49^*Aromatoleum aromaticum* (*Aa*-ADH),^49–55^ and *Bacillus stearothermophilus Ht*-ADH,^51,56^ successfully catalyzed the conversion of 1b to the corresponding hexanoic acid 4b (SI, Table S1 and Fig. S2).

We then focused on optimizing the reaction conditions. We screened three buffer systems (KPi, Tris, and HCO_3_^−^, 50 mM) at different pH values (7–10). Significant variations in enzyme performance were observed based on the buffer composition and pH. The bicarbonate buffer (HCO_3_^−^) at pH 8–9 yielded the highest formation of 4b using *Pf*-ADH, *Pp*-ADH, and *Aa*-ADH, making it the most suitable choice for the development of this biotransformation (SI, Fig. S3). However, KPi buffer performed somewhat equally well with *Pf*-ADH at the same pH values. Building on these results, we increased the concentration of 1b to 10 mM, while keeping the ADH concentration at 10 μM. While *Ht*-ADH showed reduced carboxylic acid yields at this concentration, *Pf*-ADH, *Pp*-ADH, and *Aa*-ADH retained strong aldehyde oxidation activity, achieving conversions into 4b of up to 96% (SI, Fig. S4).

**Fig. 1:**
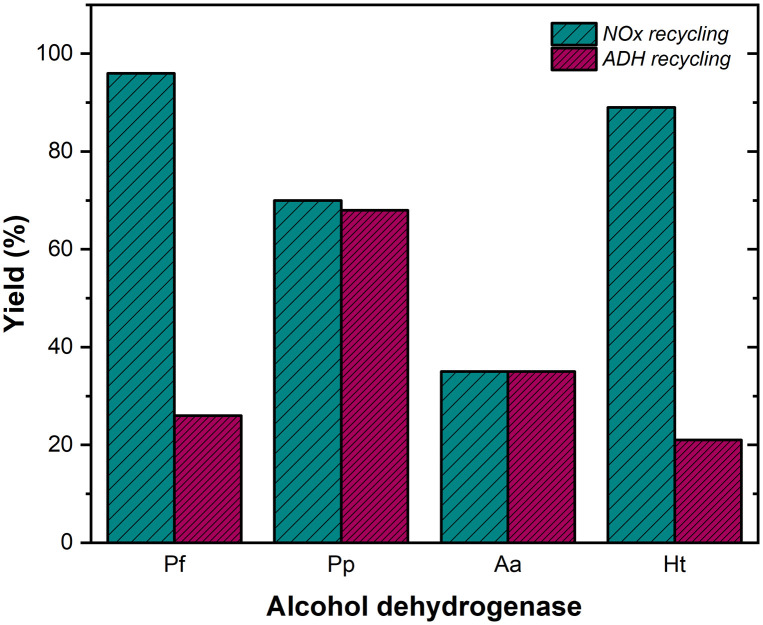
Conversion of alcohol 1b to the carboxylic acid 4b catalyzed by ADHs in the presence of an NAD^+^ cofactor recycling system *via*: a second enzyme (NOx; coupled-enzyme approach, green columns) or acetone (coupled-substrate approach, purple columns).

A known challenge in these biocatalytic oxidation processes is the low stability of NOx under the required reaction conditions.^36^ To address this issue, we explored an alternative NAD^+^ regeneration strategy that uses acetone as a sacrificial reductant (*i.e.*, the so-called coupled-substrate approach). Acetone was tested at varying concentrations (10, 20, 50, 70, and 100 eq.). The use of acetone for NAD^+^ recycling significantly reduced the conversion of 1b to carboxylic acid 4b for *Pf*-ADH and *Ht*-ADH. In contrast, *Pp*-ADH and *Aa*-ADH maintained high performance, demonstrating comparable efficiency with both acetone- and NOx-mediated cofactor recycling. The best results for *Pp*-ADH and *Aa*-ADH were achieved with 20 equivalents of acetone (Fig. 1 and SI, S5 and S6). Increasing the enzyme and cofactor concentrations also improved the conversion of 1b to the carboxylic acid 4b (SI, Fig. S7).

To explore the scalability and enhance the practical applicability of the single-enzyme biotransformation, we transitioned from using purified enzymes to cell-free extracts (CFEs, also called crude lysate, 10 mg mL^−1^). Biotransformations at 10 mM concentration of 1b with 10 mg mL^−1^ of CFE resulted in quantitative conversion (SI, Fig. S8). Furthermore, we applied a higher substrate concentration (20 mM) while maintaining the same amount of acetone (10 eq.) for NAD^+^ recycling (SI, Fig. S9). Under such conditions, *Pp*-ADH and *Aa*-ADH retained their oxidation activity from alcohol to carboxylic acid, achieving high yields comparable to those observed with purified enzymes. For example, we obtained 98% conversion of 1b to 4b for *Pp*-ADH and 62% for *Aa*-ADH at 1 mL scale in HCO_3_^−^ buffer (50 mM pH 8) with 20 mM of 1b, 10 mg of CFE of ADH and 10 eq. of acetone.

Optimization experiments assessing the influence of temperature on conversion (SI, Fig. S10) and reaction time (SI, Fig. S11) revealed that 30 °C and overnight incubation are the optimal conditions.

**Fig. 2:**
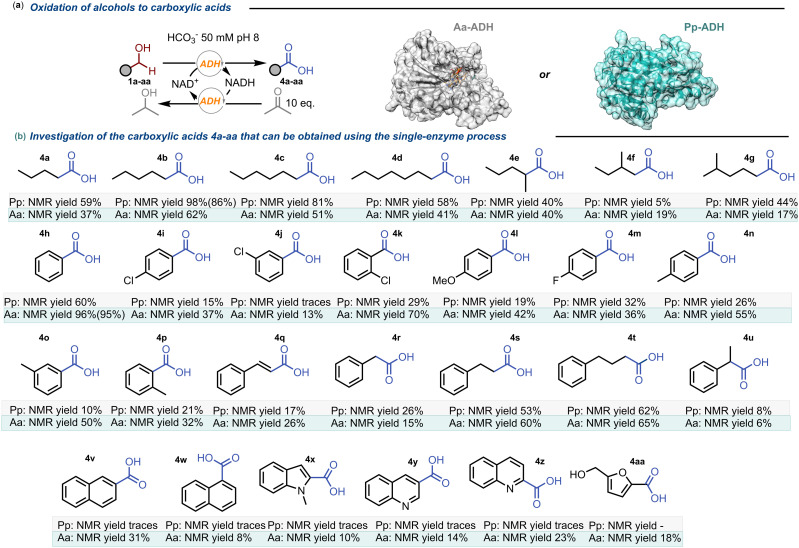
Biocatalytic reactions for the synthesis of carboxylic acids. a) General scheme of the one-enzyme redox-neutral system for the oxidation of alcohols using either *Pp*-ADH or *Aa*-ADH. b) Investigation of the carboxyl acids 4a–aa that can be obtained with the reported protocol: reactions performed using *Pp*-ADH (4 mg mL^−1^) on a 50 μmol scale of substrate 1 (20 mM) in 2.5 mL bicarbonate buffer 50 mM at pH 8.0 containing 2% DMSO. The reactions were run for 16 hours at 30 °C in an incubator equipped with temperature control. Yields are given below each entry as analytical yields (measured by NMR analysis using dioxane as the internal standard). Yields in brackets for products 4b and 4h were obtained from a scale-up using 500 mg of starting material.

To assess the scope of the biotransformation, we screened a panel of 27 structurally diverse alcohols (SI, Fig. S1) using 20 mM of substrate, ADH (CFE, 10 mg mL^−1^), acetone (10 eq., 200 mM) and NAD^+^ (0.5 mM) at 2.5 mL volume scale. A basic workup followed by an extraction step in acidic conditions, ensured the selective recovery of the carboxylic acid products in all cases without the need for any further purification. The analytical (NMR) yield of the reactions was calculated by adding dioxane as an internal standard equimolar to the substrate. Results are summarized in Fig. 2.

Notably, the two ADHs displayed complementary substrate preferences. *Pp*-ADH exhibited the highest efficiency with linear aliphatic alcohols, while aromatic substrates were generally preferred by *Aa*-ADH. The most well accepted substrate for *Pp*-ADH was found to be hexanol (1b, 98% yield) while alcohols with longer alkyl chains such as 1c and 1d were gradually less reactive. *Pp*-ADH is also a suitable catalyst for branched aliphatic substrates 1e–g, although the carboxylic acid products were obtained in moderate yields (5–44%). For substrates with aromatic substituents like benzyl alcohol 1h, *Pp*-ADH showed good yields (60%), but substitutions on the aromatic ring, particularly at the meta position (*e.g.*1j, 1o), significantly diminished the activity. Alkyl-aromatic substrates 1r–t reacted smoothly (26–62% yields), whereas bicyclic compounds (1v–z) proved far more challenging for *Pp*-ADH, leading only to traces of the corresponding carboxylic acid products.

As mentioned before, *Aa*-ADH demonstrated complementary catalytic activity towards aromatic substrates and, in general, a broader substrate acceptance. While its performance with aliphatic alcohols was moderate (17–62% yield), it consistently outperformed *Pp*-ADH on aromatic and alkyl-aromatic substrates, achieving yields of up to 96% (4h). Notably, in contrast to *Pp*-ADH, *Aa*-ADH retained activity on more sterically demanding bicyclic substrates (1v–z), producing the corresponding products with yields of up to 31%. Interestingly, for the naphthalene derivative substrates (1v and 1w), the position of the substituent influenced the catalytic activity of the enzyme which afforded 4v in 31% yield, while 4w was obtained in a much lower yield of 8%.

A particularly intriguing aspect of the *Aa*-ADH substrate scope was its different catalytic activity toward structurally related alcohols. Despite exhibiting high activity toward benzyl alcohol (1h), achieving a 96% yield of the corresponding carboxylic acid (4h), and efficiently oxidizing the bulkier substrates 3-phenyl-1-propanol (1s) and 4-phenyl-1-butanol (1t) with good yields (60% and 65%, respectively), its activity toward 2-phenylethanol (1r) was significantly lower, leading to only 15% yield. The reaction was also tested on the bio-derived substrate hydroxymethylfurfural (1aa), affording product 4aa with an 18% yield. Interestingly, the aldehyde moiety is oxidized more rapidly than the alcohol moiety. Moreover, once the carboxylic acid is formed, the alcohol moiety remains untouched, preventing the formation of dicarboxylic acids.

Finally, we performed two scale-up reactions using 1b and 1h as substrates (500 mg, each) with *Pp*-ADH and *Aa*-ADH as biocatalysts, respectively. The yields obtained in the scale-up reactions—86% and 95%, respectively—were consistent with the conversions at analytical scale—98% and 96%, respectively—demonstrating the scalability of the process. The calculated TONs for the scale-up reactions were 366 and 275, respectively. Our approach achieved a space–time yield (STY) of 0.135 g L^−1^ h^−1^, which was three to five times higher than other methods reported in the literature for the same biotransformation (see SI, section 8.4).

**Fig. 3:**
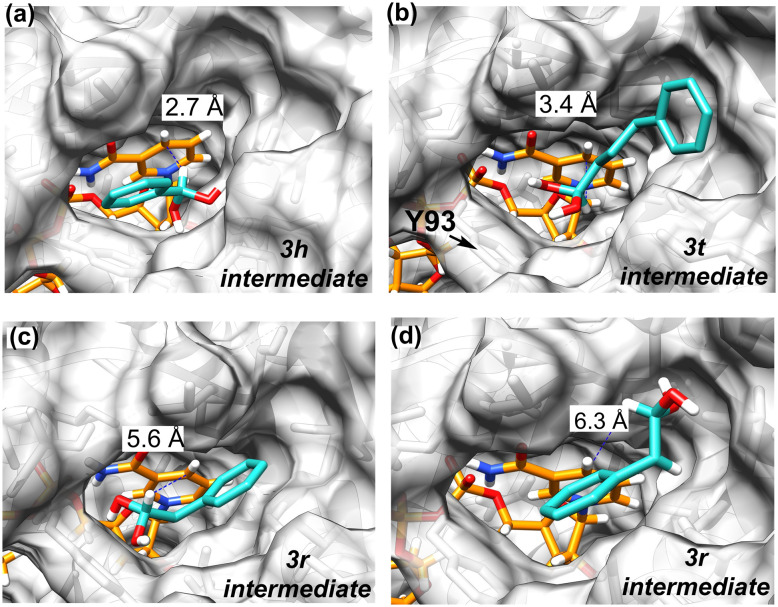
Binding poses of geminal diols as reaction intermediates in the active site of *Aa*-ADH (PDB 2EWM) identified by molecular docking: (a) phenylmethanediol (3h) with binding energy of 4.3 kcal mol^−1^ and a near-attack angle of 137°; (b) 4-phenylbutane-1,1-diol (3t) with binding energy of 4.7 kcal mol^−1^ and a near-attack angle of 93.5°; and (c and d) 2-phenylethane-1,1-diol (3r) with binding energies of 5.1 kcal mol^−1^ and a near-attack angle of 59.7° (c), as well as 4.9 kcal mol^−1^ and a near-attack angle of 68.6° (d). The NAD coenzyme in its oxidized form (NAD^+^) is shown in orange, while the substrates are depicted in light blue. Tyrosine 93 (Y93) is highlighted with an arrow due to the steric constraints it imposes on substrate binding. In each binding pose, the dashed blue line indicates the distance between the accepting carbon atom of NAD^+^ and the departing hydrogen atom of the geminal diol intermediate. Docking was performed with YASARA structure; UCSF Chimera software was used for visualization.

To rationalize our findings, we conducted molecular docking studies using the crystal structure of *Aa*-ADH with NAD^+^ (PDB 2EWM) as the receptor and substrates 1h, 1t, and 1r (Fig. S12). As the oxidation reaction proceeds through an aldehyde intermediate, which is present in equilibrium with the reactive geminal diol (aldehyde hydrate form), we also performed docking studies using the geminal diols 3h, 3t, and 3r (Fig. 3). These species are widely accepted as mechanistic intermediates, formed by nucleophilic attack of water on the carbon atom of the aldehyde moiety, a step necessary for the subsequent oxidation to carboxylic acid.^32,36,57^ Notably, this mechanism is also supported by our recent publication, in which the same ADHs were used for the synthesis of amides, thioacids and thioesters *via* hemiaminal or hemithioacetal intermediates, employing amines, hydrogen sulfide, and thiols as nucleophiles.^58^

We specifically analyzed the hydride-transfer distance between the C4 atom of the nicotinamide ring of NAD^+^ and the hydrogen atom attached to the substrate's alcohol-bearing carbon (indicated by the dashed blue line in Fig. 3 and Fig. S12), as well as the corresponding donor–hydride–acceptor angle (Cα_substrate_–H_substrate_–C4_NAD_; Table S2). These two geometric parameters define the near-attack conformation (NAC) that governs productive catalysis. In alcohol dehydrogenases, optimal NAC geometries typically feature distances of 3.0–3.5 Å and angles of 105–115°, which ensure efficient overlap of molecular orbitals.^59^

Both 3h and 3t were docked in productive binding poses with catalytically favorable orientations at distances of 2.7 Å and 3.4 Å, respectively. In contrast, 3r was found to dock in two binding poses with distances of 5.6 Å and 6.3 Å, correlating with the poor experimental conversion observed for the oxidation of 2-phenylethanol. We also performed docking of the corresponding alcohol substrates (1h, 1t, 1r, Fig. S12), which showed a similar trend, although the distance for 1h was higher than for 1t. (3.6 Å *vs.* 2.5 Å). However, the docking of the geminal diol intermediates provided a more mechanistically meaningful insight into the substrate's reactivity. Collectively, the docking studies confirm that high conversion in the oxidation of alcohols to carboxylic acids is achieved for substrates capable of adopting a favorable orientation in which the hydride donor is within an optimal distance from the acceptor carbon of the cofactor. This analysis offers a structural explanation for the reduced catalytic activity of *Aa*-ADH toward 2-phenylethanol (1r).

Notably, alcohols 1h and 1r as well as the corresponding diol intermediates 3h and 3r, were able to adopt multiple binding poses within the active site, indicating a degree of flexibility in substrate accommodation. In particular, 3h showed an alternative binding orientation, with a slightly longer distance of 3.7 Å, yet still within a tolerable range for effective hydride transfer (see Fig. S13). In contrast, the bulkier substrate 1t and its corresponding diol 3t were consistently observed in a single binding orientation. This restriction is most likely due to steric hindrance imposed by residue Y93, which constrains the available conformational space. Consequently, 1t or 3t can only bind in a fixed geometry that avoids steric clashes with Y93. While this enforced orientation still allows for productive binding, the lack of conformational freedom may limit catalytic efficiency compared to the smaller substrate 1h, which can sample multiple favorable poses.

In addition to the hydride-transfer distance, analysis of the donor–hydride–acceptor angles further supports these trends (Table S2). For 3h, despite a wider angle (137°), the short transfer distance enables efficient orbital overlap, consistent with its high conversion. 3t, with a near-optimal angle (94°), shows moderate activity, whereas 3r, characterized by both an elongated distance (>5.5 Å) and a severely distorted angle (∼60°), adopts a non-productive orientation. These geometric differences highlight that both the distance and the NAC angle jointly determine catalytic efficiency, explaining the poor catalytic activity of *Aa*-ADH toward 2-phenylethanol (1r).

Finally, to evaluate the sustainability of our methodology from a green chemistry perspective, we compared the energy consumption required for NAD^+^ recycling using NOx cell-free extracts (CFEs) *versus* acetone (SI, section 8). The energy demand was normalized for the quantitative oxidation of 1 mmol of alcohol substrate in our biotransformation. Considering the energy required for *E. coli* cell growth, cell disruption by sonication, and CFE lyophilization, the NAD^+^ recycling system *via* NOx CFE requires approximately 30 kJ per mmol of alcohol oxidized. Accounting for the energy required for acetone production *via* the cumene process and subsequent distillation, as well as its recycling through fractionation from isopropanol and extraction solvent at the end of the biotransformation, the NAD^+^ recycling system *via* acetone requires 5.6 kJ per mmol of alcohol oxidized. Therefore, NAD^+^ recycling using NOx CFE requires more than five times the energy of recycling using acetone. This comparison demonstrates the better efficiency of employing acetone as an oxidizing agent, offering a low-energy alternative to enzymatic NAD^+^ recycling. By eliminating the need for coupled-enzyme regeneration systems, this strategy better aligns with green chemistry principles and enhances the practicality of biocatalytic carboxylic acid synthesis using alcohol dehydrogenases.

## Conclusions

In this study, we developed a single-enzyme redox-neutral biocatalytic system for the oxidation of primary alcohols to carboxylic acids by harnessing the dual functionality of alcohol dehydrogenases (ADHs). By coupling alcohol and aldehyde oxidation with *in situ* NAD^+^ regeneration *via* acetone reduction, we eliminated the need for additional oxidases, significantly simplifying the reaction setup while enhancing the process's sustainability. Screening of a panel of ADHs identified two enzymes, *Pp*-ADH and *Aa*-ADH, with strong promiscuous aldehyde oxidation activity, each exhibiting distinct substrate preferences. *Pp*-ADH demonstrated superior activity with linear aliphatic alcohols, while *Aa*-ADH showed broader substrate acceptance, particularly for aromatic and alkyl-aromatic alcohols. Our findings also demonstrated the practical applicability of the process, as the use of cell-free extracts (CFEs) maintained the high conversion rates observed with purified enzymes. Moreover, the broad substrate scope, with 27 alcohols tested, confirmed the versatility of the biotransformation. Importantly, this system requires minimal workup and does not require additional purification steps, which could simplify downstream processing and reduce costs. Collectively, these features of our biocatalytic system highlight the feasibility of translating it to larger-scale applications.

From a more fundamental perspective, molecular docking studies provided insights into the substrate binding preferences of *Aa*-ADH, allowing us to rationalize its different catalytic activity with aromatic alcohols and suggesting potential directions for enhancing catalytic activities and broadening substrate scope *via* protein engineering.

Looking ahead, process intensification could enable higher efficiencies and productivities. Immobilization of the ADHs could enhance enzyme stability, allow for reuse, and improve operational robustness. Likewise, implementing the system in fed-batch or continuous-flow reactors could lead to higher space–time yields and improved cofactor turnover, ultimately enabling scalable and economically attractive biotransformations. In conclusion, this study presents a scalable, efficient, and environmentally friendly approach for the selective oxidation of alcohols to carboxylic acids, offering a competitive alternative to chemical oxidation methods.

## Experimental

### General

Materials and methods are reported in the SI, section S2. The SI also includes a list of abbreviations (section S1); materials and methods (section S2); procedures for enzyme expression and purification (section S3); details on the optimization of reaction conditions (section S4); procedures for carboxylic acid derivatization and investigation of the substrate scope (sections S5 and S6); the procedure for docking studies (section S7); greenness evaluations (section S8); and NMR spectra (section S9).

### Optimized procedure for the single-enzyme ADH-catalyzed oxidation of alcohols to carboxylic acids at 2.5 mL scale

In a 5 mL Eppendorf tube, NAD^+^ (0.5 mM) and ADH (10 mg, lyophilized cell lysate) were added to a bicarbonate buffer solution (50 mM, pH 8) to a final volume of 2.5 mL. Acetone (10 eq.) was added for NAD^+^ cofactor recycling. The alcohol substrate was added last from a 1 M stock solution in DMSO, resulting in a final substrate concentration of 20 mM. The reaction mixture was incubated at 30 °C and agitated at 170 rpm on an orbital shaker for 24 hours. After incubation, the aqueous phase was basified and extracted with EtOAc (3 × 1 mL) to remove unreacted starting material and any possible aldehyde by-product. The aqueous phase was then acidified to pH 2 using 2 M HCl and extracted again with EtOAc (3 × 1.5 mL). The combined organic layers were dried over anhydrous MgSO_4_ and analyzed by ^1^H NMR using 1,4-dioxane as an internal standard to determine the yield.

### Preparative-scale single-enzyme ADH-catalyzed oxidation of alcohols to carboxylic acids

In an Erlenmeyer flask (500 mL), NAD^+^ (0.5 mM), ADH (10 mg mL^−1^ cell-free extract), and acetone (200 mM) were added to a bicarbonate buffer solution (50 mM, pH 8) to a final volume of 245 mL or 230 mL for scale-up reactions with substrates 1b and 1h, respectively. The substrate (500 mg, 20 mM) was added from a DMSO stock solution, resulting in a final DMSO concentration of 1% (v/v) in the reaction mixture. The reaction was incubated at 30 °C and agitated at 170 rpm for 24 h on an orbital shaker. Upon completion, the reaction mixture was basified with 2 M NaOH and extracted with ethyl acetate (3 × 100 mL). The aqueous phase was then acidified to pH 2 with 4 M HCl and extracted again with ethyl acetate (3 × 100 mL). The combined organic layers were dried over anhydrous MgSO_4_, concentrated under reduced pressure, and analyzed by ^1^H NMR. The carboxylic acid products 4b and 4h were obtained in chemically pure form with isolated yields of 86% and 95%, respectively.

## Author contributions

F. G. M. and M. D. conceived the project. M. D. and Z. W. performed the wet lab experiments and analyzed the data. V. T. performed the computational studies. F. G. M. acquired the funding and supervised project. The manuscript was written through contribution of all the authors and final version was approved by all the authors.

## Conflicts of interest

The authors declare to have no competing interests, or other interests that might be perceived to influence the results and/or discussion reported in this article.

## Supplementary Material

CY-016-D5CY01223F-s001

## Data Availability

Supplementary information is available. See DOI: https://doi.org/10.1039/d5cy01223f.
