# Supplementary material for: Single-enzyme redox-neutral oxidation of alcohols to carboxylic acids using alcohol dehydrogenases
Source: Catal Sci Technol. 2025 Dec 10;16(3):876–83. doi: 10.1039/d5cy01223f (PMC12775759; doi:10.1039/d5cy01223f)
Supplement: CY-016-D5CY01223F-s001 [file CY-016-D5CY01223F-s001.pdf]

Supplementary Information (SI) for Catalysis Science & Technology.
This journal is © The Royal Society of Chemistry 2025

Supporting Information

For

Single-Enzyme Redox-Neutral Oxidation of Alcohols to Carboxylic Acids Using Alcohol Dehydrogenases

Matteo Damian, [‡a] Zheng Wei, [‡a] Vasilis Tseliou[a] and Francesco G. Mutti *[a]

Van 't Hoff Institute for Molecular Sciences, HIMS-Biocat, University of Amsterdam, Science Park 904, 1098 XH, The Netherlands.

*Corresponding author, e-mail: f.mutti@uva.nl

[‡] These authors contributed equally to this work.

Contents

1. List of abbreviations ... 3
2. Material and methods ... 3
   2.1 List of substrates ... 3
   2.2 List of enzymes ... 4
3. General procedure for enzymes expression and purification ... 5
   3.1 Enzyme expression ... 5
   3.2 Purification by Nickel ion affinity chromatography ... 5
4. Optimization ... 7
   4.1 First screening ... 7
   4.2 Testing at higher substrate loadings in optimal buffers ... 8
   4.3 Testing of acetone for cofactor recycling ... 8
   4.4 Optimization of acetone concentration in different buffers ... 9
   4.5 Testing at higher amount of enzyme (Pp-ADH) and cofactor ... 10
   4.6 Testing of cell free extract (CFE) ... 10
   4.7 Testing at higher substrate loading using cell free extract (CFE) ... 11
   4.8 Temperature screening ... 12
   4.9 Time study ... 13
5. Derivatization ... 14
6. Optimized reaction conditions used to investigate the substrate scope ... 14
7. Docking studies ... 14
8. Greenness evaluations ... 17
   8.1 E factor determination ... 17
   8.2 Calculation for NAD^+ cofactor recycling using NOx CFE ... 18
   8.3 Calculation for NAD^+ cofactor recycling using acetone ... 19
   8.4 Calculation for productivity (Space-time-yield) ... 19
9. NMR spectra ... 19
10. References ... 38

1. List of abbreviations

ADH	Alcohol dehydrogenase
NOx	Nicotinamide adenine dinucleotide oxidase
NAD(P)$^+$	Nicotinamide adenine dinucleotide or its phosphate analogue (oxidized form)
GC-FID	Gas chromatography with flame ionization detector
GC-MS	Gas chromatography coupled with mass spectrometry
GOx	Galactose oxidase
E. coli	*Escherichia coli*
CFE	Cell free extract

2. Material and methods

Nicotinamide adenine dinucleotide cofactors (NAD$^+$ or NADP$^+$) were purchased from Melford Biolaboratories (Chelsworth, Ipswich, UK).

The conversions during the optimization stage were determined by GC using a 7890A GC system (Agilent Technologies), equipped with an FID detector using H_2 as carrier gas and a DB-1701 column from Agilent (30 m, 250 µm, 0.25 µm).

^{1}H (400 MHz) spectra were recorded at ambient temperature using a Bruker AV400. ^{1}H NMR spectra are reported in parts per million (ppm) downfield relative to $CDCl_3$ (7.26 ppm). NMR data were processed using the MestReNova 14.1.0 software package.

2.1 List of substrates

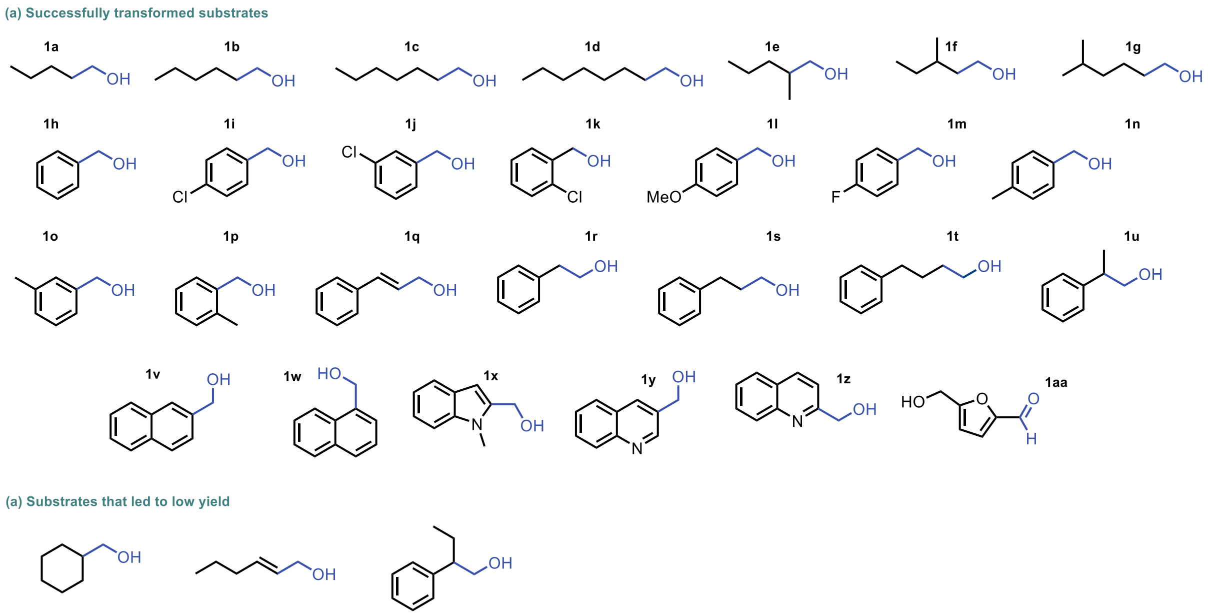

Figure S1: The primary alcohols used in the current work.

2.2 List of enzymes

Table S1: Enzymes used in this work.

**Abbreviation**	**Source**	**Selectivity**	**Cofactor**	**Used form**	**Ref**
Pf-ADH	ADH from *Pichia finlandica*	Anti-Prelog	NAD	Purified	[1]
Aa-ADH	ADH from *Aromatoleum aromaticum*	Prelog	NAD	Purified and crude lysate	[2]
Pp-ADH	ADH from *Paracoccus pantotrophus* DSM 11072	Prelog	NAD	Purified and crude lysate	[3]
Ht-ADH	ADH from *Bacillus stearothermophilus*	Primary alcohols	NAD	Purified	[4]
NOx	NOx from *Streptococcus mutans*	n. a.	n. a.	Purified	[5]

3. General procedure for enzymes expression and purification

3.1 Enzyme expression

For recombinant expression, 800 mL of LB medium supplemented with the appropriate antibiotic (100 µg mL^{-1} ampicillin or 50 µg mL^{-1} kanamycin) were inoculated with 15 mL of an overnight culture harboring the desired vector with genes for the expression of the enzyme. *E. coli* BL21(DE3) cells were used as expression host organism in this study. Cells were grown at 37 °C until an OD_{600} in the range from 0.6 to 1 was reached and expression of protein was induced by the addition of IPTG. Enzyme expression was carried out overnight and after harvesting of the cells (4 °C, 4500 rpm, 10 min), the remaining cell pellets were washed with buffer. For the preparation of lyophilized *E. coli* whole cells, we used: 50 mM KPi buffer at pH 8.0 for ADHs and NOx. For the preparation of the cell lysate for further enzyme purification by affinity chromatography, we used lysis buffer as reported in the next paragraph.

3.2 Purification by Nickel ion affinity chromatography

His_6-tagged enzymes were resuspended in lysis buffer (50 mM KH_2PO_4, 300 mM NaCl, 10 mM imidazole, pH 8.0, 5 mL per 1 g of wet cells) prior to cell disruption via sonication (10 min, pulse 10 s ON, pulse 10 s OFF, 45% amp). Before the disruption of cells containing overexpressed NOx, a small amount of FAD was externally added. Enzyme purification was performed by Ni-NTA affinity chromatography using pre-packed Ni-NTA HisTrap HP columns (GE Healthcare), previously equilibrated with lysis buffer. After loading of the filtered lysate, the column was washed with sufficient amounts of washing buffer (50 mM KH_2PO_4, 300 mM NaCl, 25 mM imidazole, pH 8.0), and bound protein was recovered with elution buffer (50 mM KH_2PO_4, 300 mM NaCl, 200 mM imidazole, pH 8.0). Enzymes purity was analyzed by SDS-PAGE and fractions containing the enzyme were combined and dialyzed overnight against KPi buffer (4 L, pH 8.0, 50 mM). The enzyme solutions were concentrated, and their concentration was determined spectrophotometrically based on their extinction coefficient at the wavelength of 280 nm. The concentration of NOx enzyme was determined based on the extinction coefficient of FAD at the wavelength of 440 nm.

Yield of production:

Pf-ADH: 22 $mg_{enzyme}/g_{wet\ cells}$
115 $mg_{enzyme}/L_{LB\ medium}$

Pp-ADH: 14 $mg_{enzyme}/g_{wet\ cells}$
74 $mg_{enzyme}/L_{LB\ medium}$

Aa-ADH: 20 $mg_{enzyme}/g_{wet\ cells}$
101 $mg_{enzyme}/L_{LB\ medium}$

Ht-ADH: 30 $mg_{enzyme}/g_{wet\ cells}$
183 $mg_{enzyme}/L_{LB\ medium}$

NOx: 13 $mg_{enzyme}/g_{wet\ cells}$
70 $mg_{enzyme}/L_{LB\ medium}$

SDS-Page

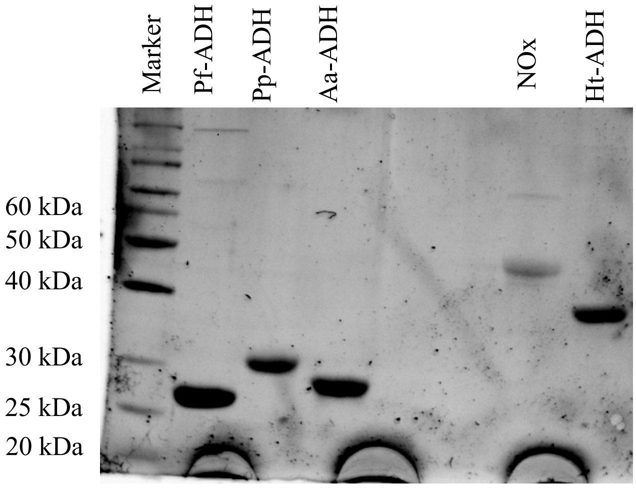

Figure S2: SDS-PAGE of purified enzymes. Marker: PageRuler™ Unstained Protein Ladder (ThermoFisher Scientific).

4. Optimization

4.1 First screening

In an Eppendorf tube (2 mL), NAD^+ (0.5 mM), NOx (10 µM) and the ADH (10 µM) were added in the appropriate buffer solution (varied concentration and pH, final volume of 1 mL). The hexanol substrate (**1b**) was added from a 1 M DMSO stock solution as last to have a final substrate concentration in the solution of 5 mM. The reaction was incubated at 30 °C, at 170 rpm for 24 h on an orbital shaker. Then, the aqueous phase was acidified to pH 2 with HCl 2 M and was extracted with ethyl acetate (500 µL x 2). The organic layer was dried over $MgSO_4$ and analyzed by GC-FID on DB-1701 30 m column after derivatization to the corresponding methyl esters. Measurements are the average of at least three independent replicates.

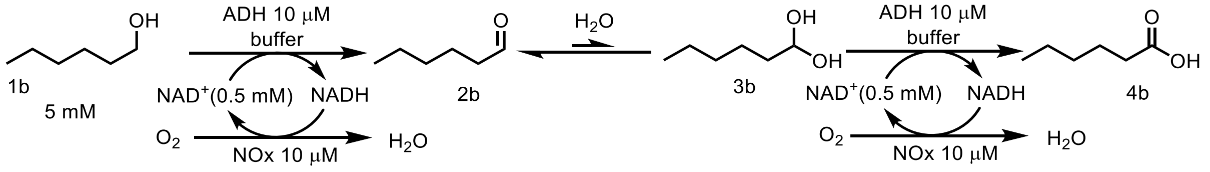

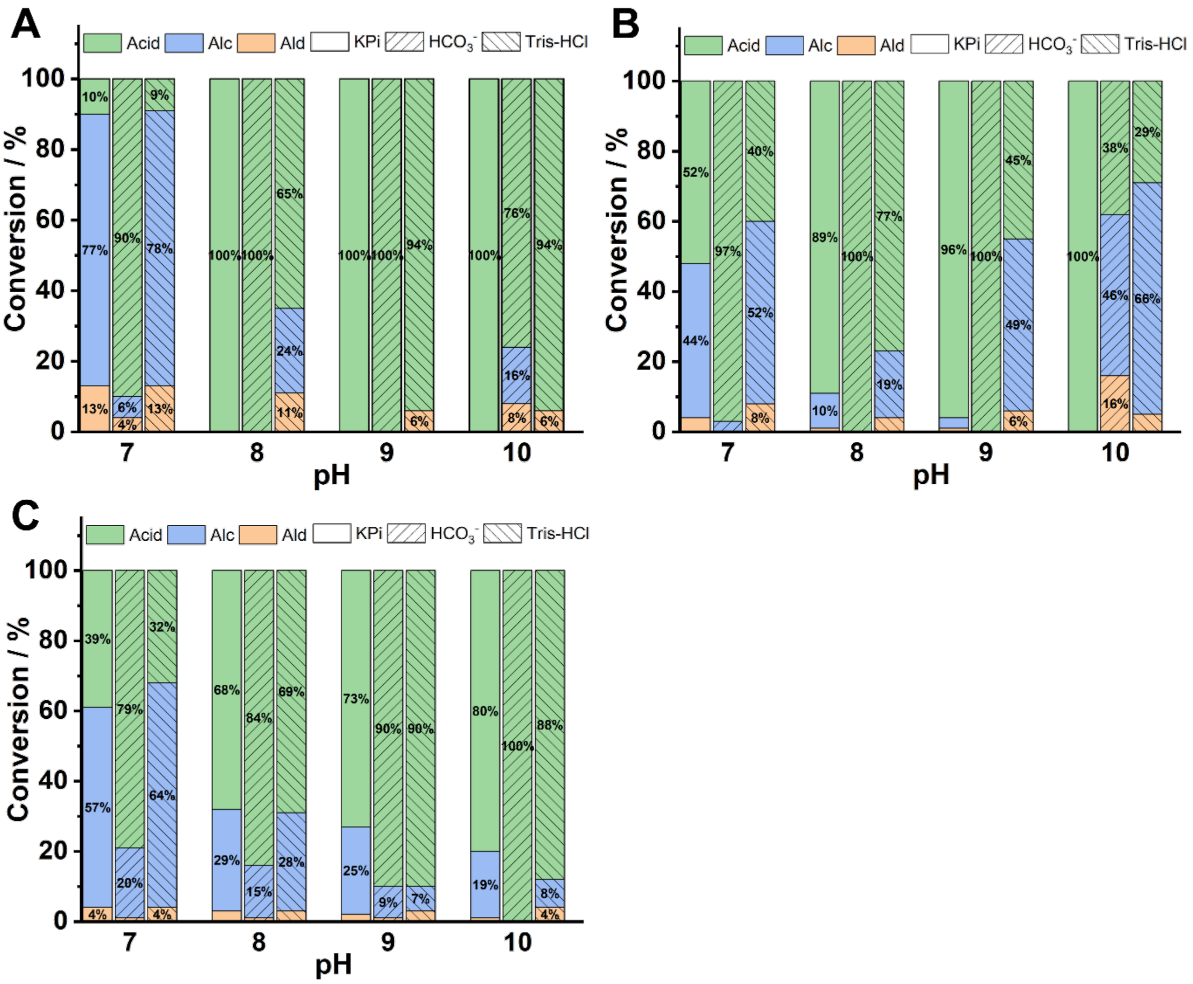

Figure S3. The conversion of alcohol **1b** to the corresponding aldehyde **2b** and carboxylic acid **4b** catalyzed by Pf-ADH (A), Pp-ADH (B) and Aa-ADH (C) in different buffer solutions (pH 7–9).

4.2 Testing at higher substrate loadings in optimal buffers

In an Eppendorf tube (2 mL), NAD^+ (0.5 mM), NOx (10 µM) and ADH (10 µM) were added in the different buffers (50 mM varied pH, final volume of 1 mL). The substrate was added from a 1 M DMSO stock solution as last having a final concentration of 10 mM. The reaction was incubated at 30 °C, 170 rpm for 24 h on an orbital shaker. Then, the aqueous phase was acidified to pH 2 with HCl 2 M and was extracted with ethyl acetate (500 µL x 2). The organic layer was dried over $MgSO_4$ and analyzed by GC-FID on DB-1701 30 m column after derivatization to the corresponding methyl esters. During the study, each point of on any graph was at least the average value obtained from three independent tests.

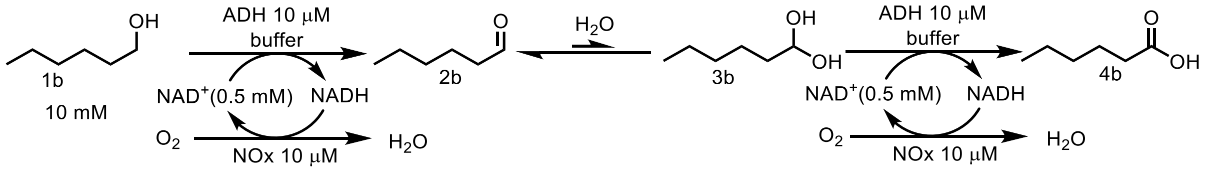

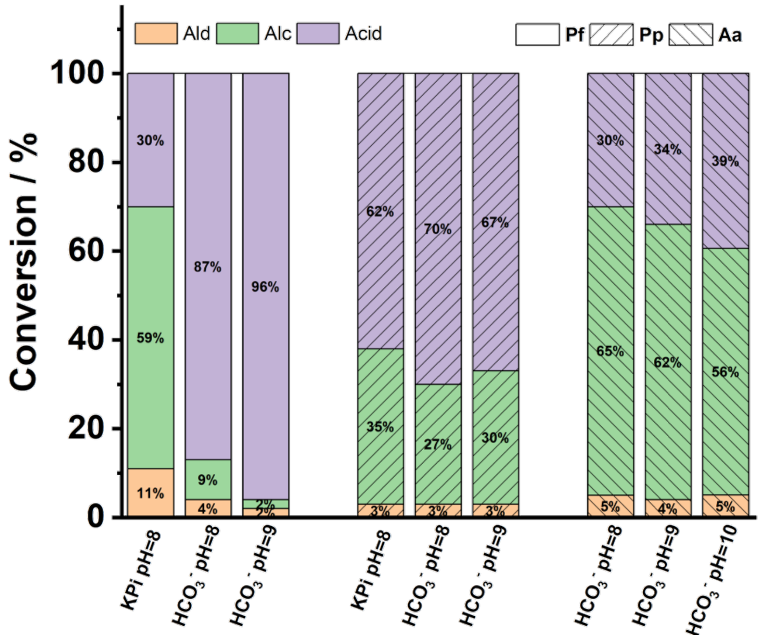

Figure S4. Investigation of biocatalytic conversion of alcohol **1b** to corresponding aldehyde **2b** and carboxylic acid **4b** catalyzed by Pf-ADH, Pp-ADH and Aa-ADH in KPi buffer (50 mM, pH 8) or HCO_3^- buffer (50 mM, pH 8 or 9) at higher substrate loading (10 mM).

4.3 Testing of acetone for cofactor recycling

In an Eppendorf tube (2 mL), NAD^+ (0.5 mM), and ADH (10 µM) were added in a total of 1 mL HCO_3^- (50 mM, pH 8). Instead of NOx, acetone was added to test the different approach for cofactor recycling. The substrate was added from a 1 M DMSO stock solution to have a final concentration of 10 mM. The reaction was incubated at 30 °C, 170 rpm for 24 h on an orbital shaker. Then, the aqueous phase was acidified to pH 2 with HCl 2 M and was extracted with ethyl acetate (500 µL x 2). The organic layer was dried over $MgSO_4$ and analyzed by GC-FID on DB-1701 30 m column after derivatization to the corresponding methyl esters. Measurements are the average of three independent replicates.

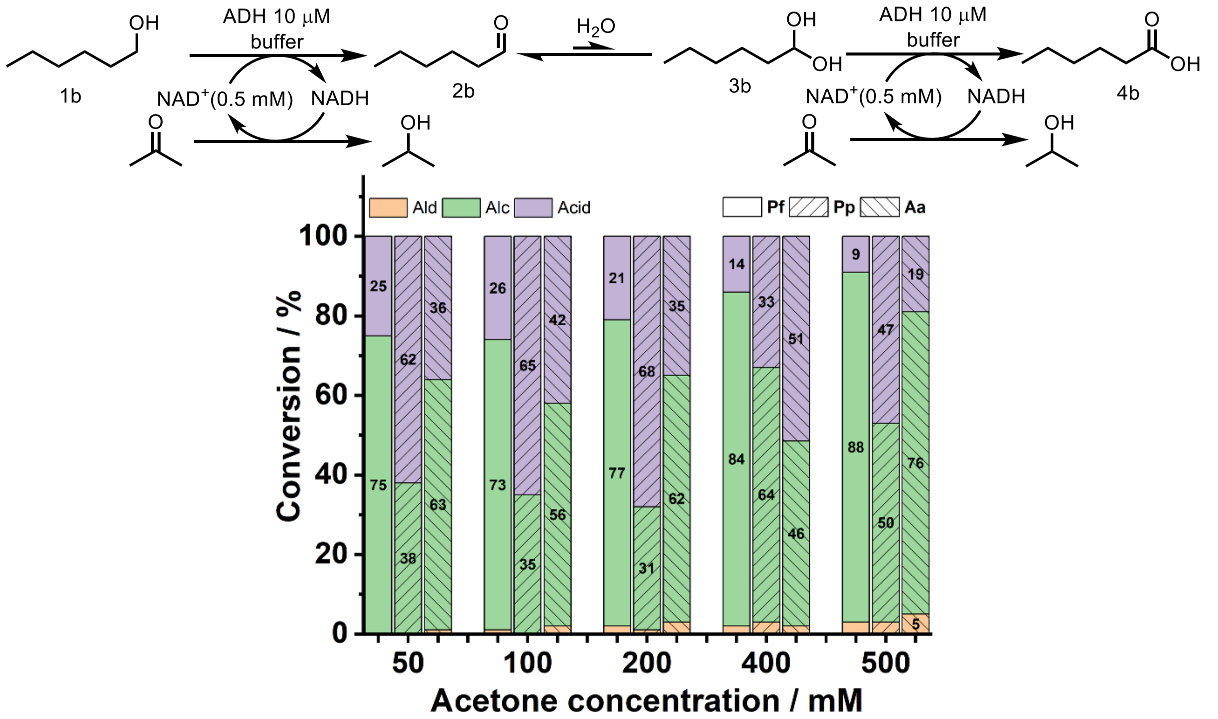

Figure S5. Conversion of alcohol **1b** to corresponding aldehyde **2b** and carboxylic acid **4b** catalyzed by Pf-ADH, Pp-ADH, and Aa-ADH using acetone as NAD+-recycling system.

4.4 Optimization of acetone concentration in different buffers

In an Eppendorf tube (2 mL), NAD+ (0.5 mM), and ADH (10 μM) were added in the best performing buffer (i.e, HCO_3^- 50 mM and pH 8 and 9, final volume of 1 mL). Instead of adding NOx, acetone was added at different concentrations. The substrate was added from a 1 M DMSO stock solution as last to have the final concentration of 10 mM. The reaction was incubated at 30 °C, 170 rpm for 24 h on an orbital shaker. Then, the aqueous phase was acidified to pH 2 with HCl 2 M and was extracted with ethyl acetate (500 μL x 2). The organic layer was dried over $MgSO_4$ and analyzed by GC-FID on DB-1701 30 m column after derivatization to the corresponding methyl esters. Measurements are the average of three independent replicates.

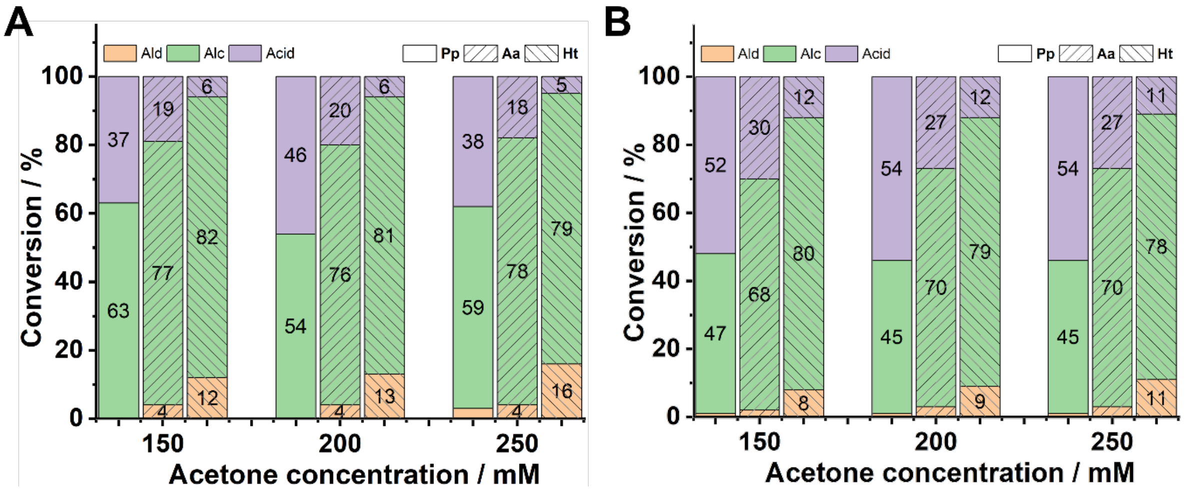

Figure S6. The conversion of alcohol **1b**, to the corresponding aldehyde **2b**, and carboxylic acid **4b** catalyzed by Pp-ADH, Aa-ADH, and Ht-ADH using different concentration of acetone for NAD+ regeneration in 50 mM HCO_3^- buffer solution at pH=9 (A) and pH=8 (B).

4.5 Testing at higher amount of enzyme (Pp-ADH) and cofactor

In an Eppendorf tube (2 mL), NAD^+ (0.5–1 mM), Pp-ADH (10–20 µM) and acetone (20 eq, 200 mM) were added in the 50 mM HCO_3^- pH 8, to a final volume of 1 mL. The substrate was added last from a 1 M DMSO stock solution to have the final concentration of 10 mM. The reaction was incubated at 30 °C, 170 rpm for 24 h on an orbital shaker. Then, the aqueous phase was acidified to pH 2 with HCl 2 M and was extracted with ethyl acetate (500 µL x 2). The organic layer was dried over $MgSO_4$ and analyzed by GC-FID on DB-1701 30 m column after derivatization to the corresponding methyl esters. Measurements are the average of three independent replicates.

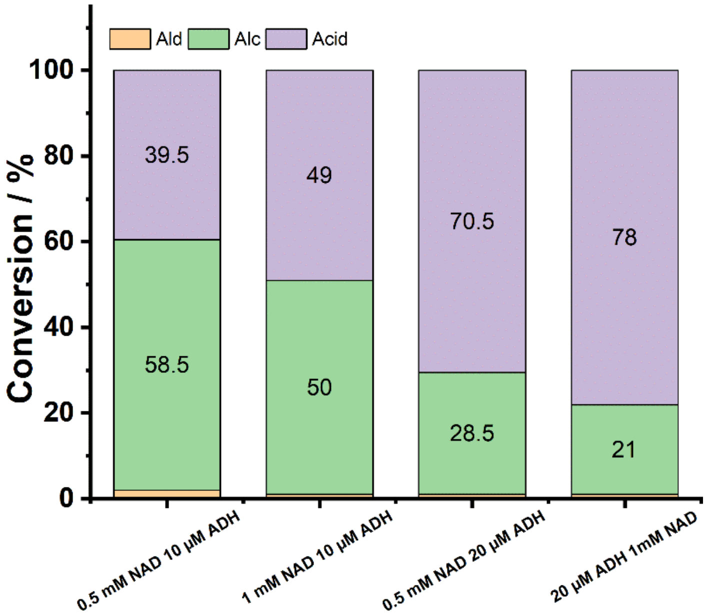

Figure S7. The conversion of alcohol **1b**, to the corresponding aldehyde **2b**, and carboxylic acid **4b** catalyzed by Pp-ADH with different concentrations of NAD^+ and ADHs.

4.6 Testing of cell free extract (CFE)

In an Eppendorf tube (2 mL), NAD^+ (0.5–1 mM), Pp-ADH as lyophilized cell free extract (5, 10 or 15 mg), and acetone (200 mM) were added in 50 mM HCO_3^-pH 8, until the final volume of 1 mL. The substrate was added last from a 1 M DMSO stock to have the final concentration of 10 mM. The reaction was incubated at 30 °C, 170 rpm for 24 h on an orbital shaker. Then, the aqueous phase was acidified to pH 2 with HCl 2 M and was extracted with ethyl acetate (500 µL x 2). The organic layer was dried over $MgSO_4$ and analyzed by GC-FID on DB-1701 30 m column after derivatization to the corresponding methylesters. Measurements are the average of three independent replicates.

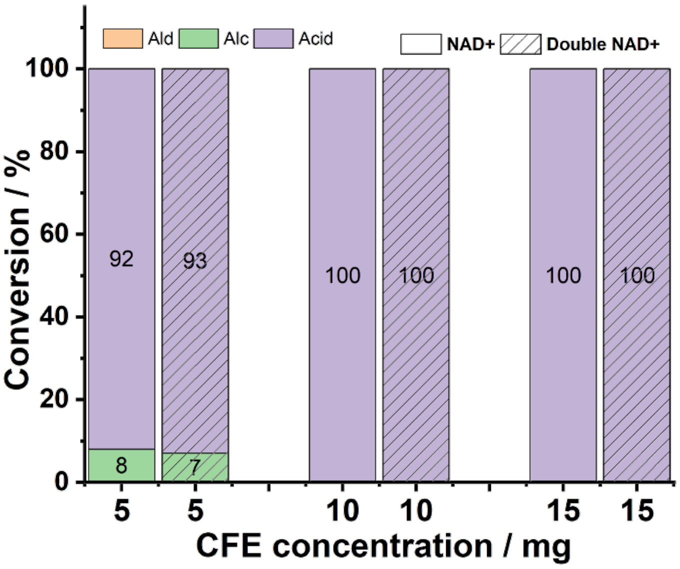

Figure S8. The conversion of alcohol **1b**, to the corresponding aldehyde **2b**, and carboxylic acid **4b** catalyzed by different amounts of CFE of Pp-ADH and at different concentrations of NAD^+.

4.7 Testing at higher substrate loading using cell free extract (CFE)

In an Eppendorf tube (2 mL), NAD^+ (0.5 mM), Pp-ADH as lyophilized cell free extract (5, 10 or 15 mg) and acetone (200 mM) were added in 50 mM HCO_3^- pH 8, until the final volume of 1 mL. The substrate was added last from a 1 M DMSO stock solution to have a final concentration of 20, 30 or 50 mM. The reaction was incubated at 30 °C, 170 rpm for 24 h on an orbital shaker. Then, the aqueous phase was acidified to pH 2 with HCl 2 M and was extracted with ethyl acetate (500 µL x 2). The organic layer was dried over $MgSO_4$ and analyzed by GC-FID on DB-1701 30 m column after derivatization to the corresponding methyl esters. During the study, each point of on any graph was at least the average value obtained from three independent tests.

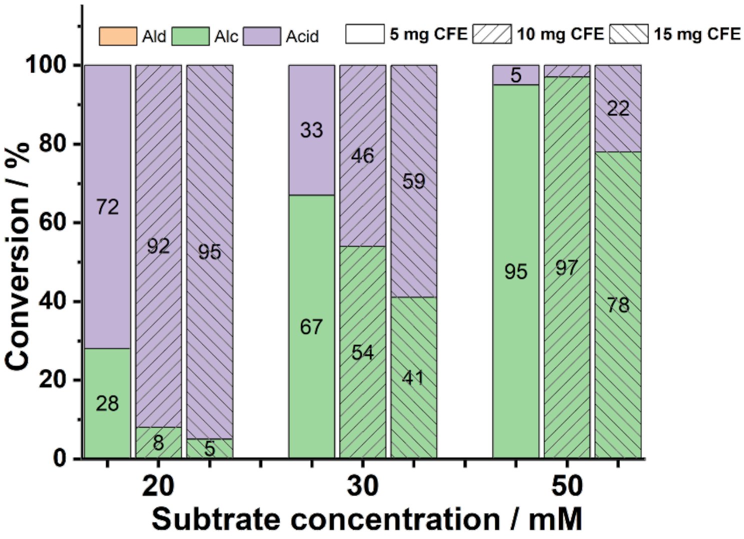

Figure S9. The conversion of alcohol **1b**, to the corresponding aldehyde **2b**, and carboxylic acid **4b** catalyzed by different amounts of CFE of Pp-ADH and at different substrate concentrations.

4.8 Temperature screening

In an Eppendorf tube (2 mL), NAD^+ (0.5 mM), 10 mg of Pp-ADH as lyophilized cell free extract and acetone (200 mM) were added in 50 mM HCO_3^- pH 8, until the final volume of 1 mL. The substrate was added last from a 1 M DMSO stock solution to the final concentration of 20 mM. The reaction was incubated at different temperatures, 170 rpm for 24 h on an orbital shaker. Then, the aqueous phase was acidified to pH 2 with HCl 2 M and was extracted with ethyl acetate (500 µL x 2). The organic layer was dried over $MgSO_4$ and analyzed by GC-FID on DB-1701 30 m column after derivatization to the corresponding methylesters. During the study, each point of on any graph was at least the average value obtained from two independent tests.

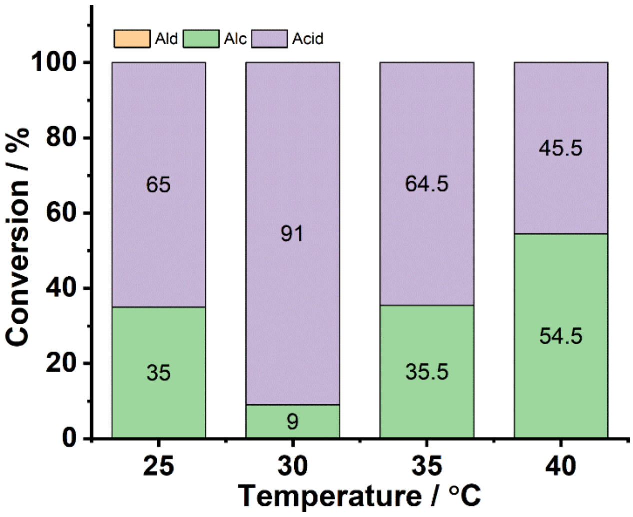

Figure S10. The conversion of alcohol **1b**, to the corresponding aldehyde **2b**, and carboxylic acid **4b** catalyzed by CFE of Pp-ADH at different temperatures.

4.9 Time study

In an Eppendorf tube (2 mL), NAD^+ (0.5 mM), 10 mg of Pp-ADH as lyophilized cell free extract and acetone (200 mM) were added in 50 mM HCO_3^- pH 8, until the final volume of 1 mL. The substrate was added last to the final concentration of 20 mM. The reaction was incubated at 30 °C, 170 rpm for 24 h on an orbital shaker. Then, the aqueous phase was acidified to pH 2 with HCl 2 M and was extracted with ethyl acetate (500 µL x 2). The organic layer was dried over $MgSO_4$ and analyzed by GC-FID on DB-1701 30 m column after derivatization to the corresponding methylesters. During the study, each point of on any graph was at least the average value obtained from two independent tests.

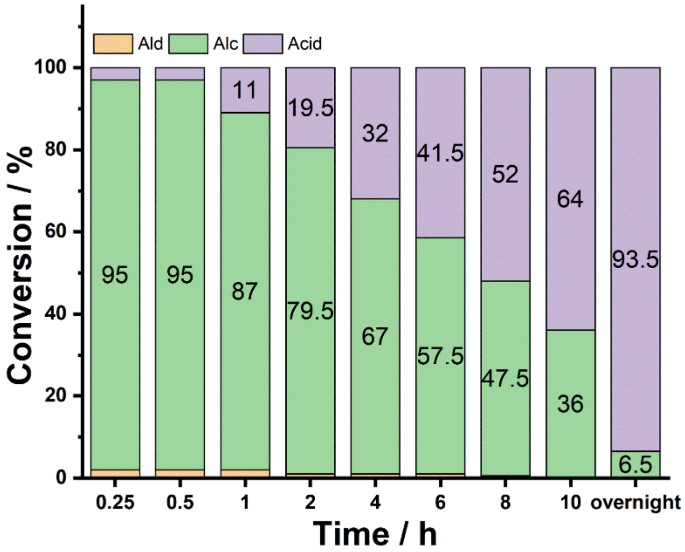

Figure S11. The conversion of alcohol **1b**, to the corresponding aldehyde **2b**, and carboxylic acid **4b** catalyzed by CFE of Pp-ADH at different reaction times.

5. Derivatization

The analytical scale biotransformations were extracted with ethyl acetate (total volume 1 mL). An aliquot of this organic phase (250 µL) was combined with a solution of EtOAc (540 µL), MeOH (200 µL) and (trimethylsilyl)diazomethane (10 µL). The resulting 1 mL reaction was shaken at 30 °C, 160 rpm for 60 min. The excess of the derivatization reagent was quenched by the addition of acetic acid (2 µL) and by incubating for further 30 min at 30 °C. Analysis was performed by GC-FID.

6. Optimized reaction conditions used to investigate the substrate scope

In an Eppendorf tube (5 mL), NAD^+ (0.5 mM), and ADHs (10 mg CFE) were added in the HCO_3^- buffer (50 mM, pH 8) to a final volume of 2.5 mL. Acetone (10 eq) was added for NAD^+ cofactor recycling. Alcohol substrate was added from a 1 M DMSO stock solution as last to have a final concentration in the solution of 20 mM. The reaction was incubated at 30 °C, 170 rpm for 24 h on an orbital shaker. Then, the aqueous phase was basified and extracted three times (1 mL) with EtOAc to remove starting material and by-products. Afterwards, the aqueous phase was acidified to pH 2 with HCl 2 M and was extracted with ethyl acetate (1 mL x 3). The organic layer was dried over $MgSO_4$ and analyzed by ^1H NMR, using an internal standard (1,4 dioxane) to determine the yield.

7. Docking studies

The molecular dockings were performed using Autodock Vina as tool incorporated into YASARA using the crystal structure of Aa-ADH in complex with NAD (PDB: 2EWB). The substrate molecules (alcohols and their corresponding geminal-diol intermediates) were constructed in YASARA and energy-minimized with the AMBER03 force field.[6] In all simulations, a cubic docking cell was positioned to enclose the catalytic cavity, with its center placed on the C4 atom of the nicotinamide ring of NAD^+ (C4N, atom ID 1802) and extending 10 Å in each direction.

The receptor was kept rigid during all calculations. Each ligand was subjected to 25 independent AutoDock Vina runs (exhaustiveness = 8). After clustering the resulting poses at a 5.0 Å RMSD cutoff, the most representative conformations were selected based on their binding energy and productive orientation toward the NAD^+ cofactor. In all cases, higher (more positive) binding-energy values indicate stronger binding, whereas negative values correspond to non-productive or repulsive poses.

For each geminal-diol intermediate, the top-ranked docking clusters are reported in Table S2, together with the corresponding near-attack conformation (NAC) parameters and hydride-transfer distances. All final poses were inspected visually to confirm chemically meaningful orientations within the active site.

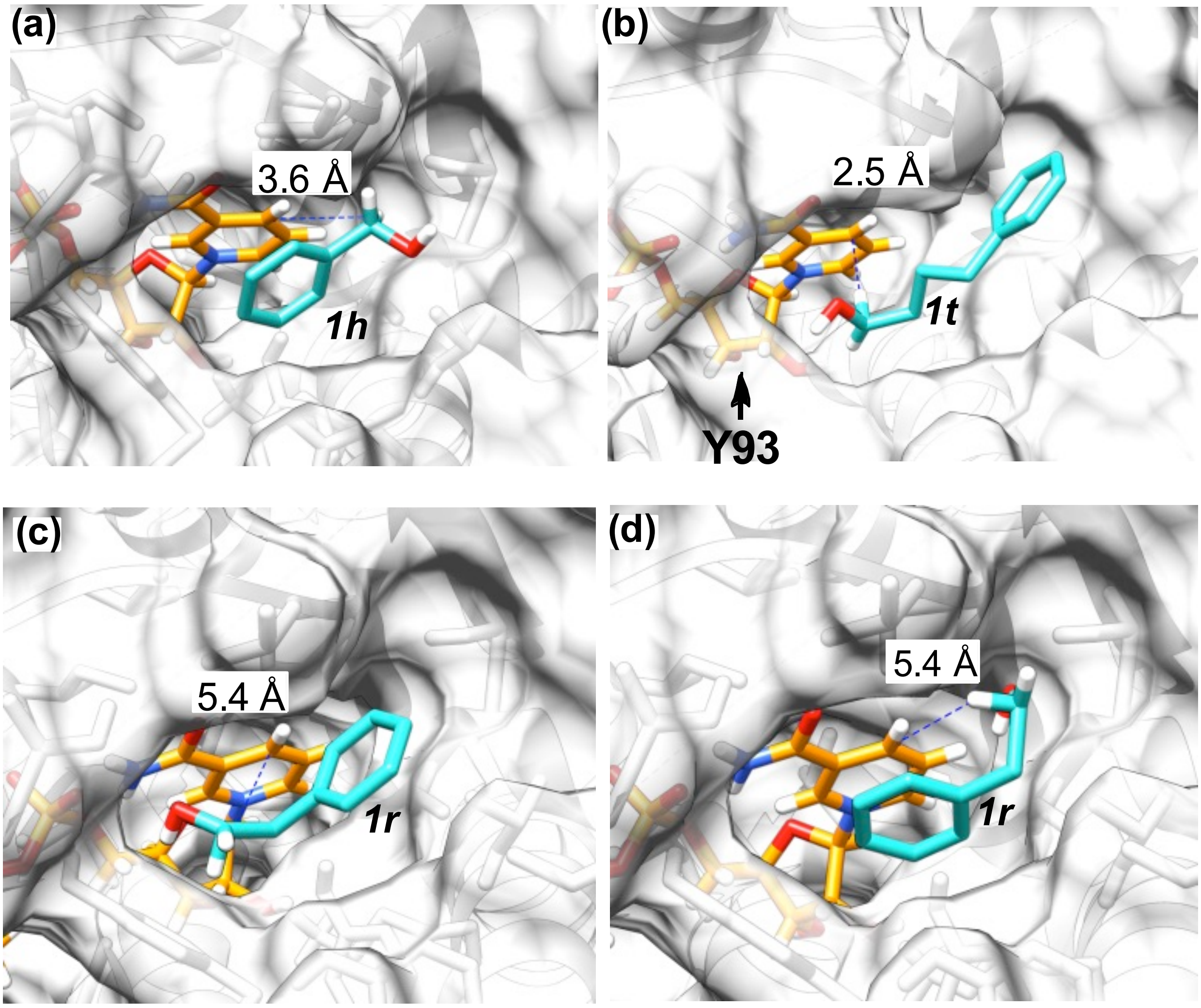

Figure S12. Binding poses in the active site of Aa-ADH (PDB 2EWM) identified by molecular docking: (a) benzyl alcohol (**1h**) with binding energy of 4.6 Kcal mol^{-1}; (b) 4-phenyl-1-butanol (**1t**) with binding energy of 4.8 Kcal mol^{-1}; and (c,d) 2-phenyl-ethanol (**1r**) with binding energy of 4.7–4.8 Kcal mol^{-1}). The NAD coenzyme in its oxidized form (NAD^+) is shown in orange, while the substrates are depicted in light blue. Tyrosine 95 (Y95) is highlighted with an arrow due to the steric constraints it imposes on substrate binding. In each binding pose, the dashed blue line indicates the distance between the accepting carbon atom of NAD^+ and the departing hydrogen atom of the alcohol substrate. Dockings performed with YASARA structure; UCSF Chimera software was used for visualization.

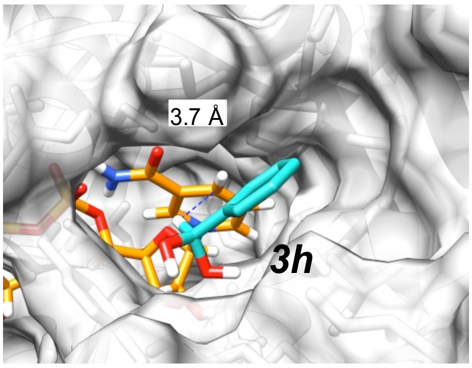

Figure S13. Alternative binding pose of phenylmethanediol (**3h**) in the active site of Aa-ADH identified by molecular docking with binding energy of 4.4 Kcal mol^{-1}.

Table S2. Docking results and calculated near-attack conformation (NAC) parameters for geminal-diol intermediates docked in Aa-ADH.

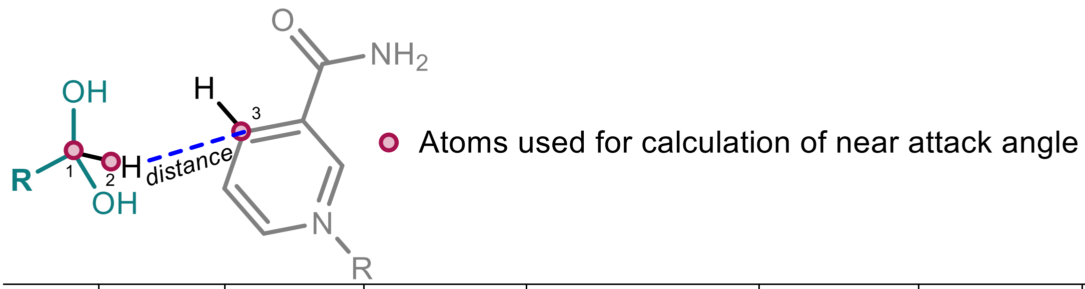

Entry	Enzyme	Substrate	Docking clusters	Binding energy [kcal mol^{-1}]	Near-attack angle [°]	Distance H_{sub}-C_{NAD} [Å]	Ref
1	Aa-ADH	**3h**	**001**	**000004.2600**	**137.033**	**2.7**	**Fig. 3a**
			002	000004.6850	40.267	6.2	
			003	000003.3780	78.013	5.7	
2	Aa-ADH	**3t**	**001**	**000004.6730**	**93.552**	**3.4**	**Fig. 3b**
			002	000003.9710	102.020	9.0	
			003	000003.7380	21.280	4.6	
3	Aa-ADH	**3r**	**001**	**000005.0700**	**59.726**	**5.6**	**Fig. 3c**
			**002**	**000004.9060**	**68.600**	**6.3**	**Fig. 3d**
			003	000003.4750	40.905	7.6	

8. Greenness evaluations

8.1 E factor determination

We selected the two most representative papers for the single enzyme catalyzed formation of carboxylic acids starting from alcohols: the work published by Turner in 2018[7] and the one from Grogan in 2025[8], using Galactose Oxidase (GOx) and Unspecific peroxygenases (UPOs), since the other papers were presenting mostly preliminary data on very specific substrates and with low conversions.
Both works are using lower substrate loading than our work (10 and 12.5 mM, respectively) and, while the UPOs are used as cell-free extract, the GOx have been purified, meaning that a large volume of buffer, and related salts, has been used for this purpose, making the whole process less sustainable.
Taking into account only the reaction per se, without considering the whole process for the preparation of the enzyme, we evaluated the E factor of our reaction, with the example of Benzyl alcohol, and compared to the one from Turner with the same substrate and the one from Grogan with cinnamyl alcohol.
Our E factor turned out to be the lowest; this result, together with the broad applicability of the method, highlights the efficiency and versatility of our approach.

This work: Benzyl alcohol to benzoic acid
96% yield = 5.4 mg

Table S3: Reaction table for our work to evaluate the waste generated and the E factor.

Reagents	MW [g/mol]	Density [g/mL]	µmol	Mass [mg]	Volume [µL]
Benzyl alcohol	108.1	1.044	50	5.407	5.179
ADH	N/A	N/A	N/A	250	N/A
NAD^+	680.4	N/A	1.25	0.85	N/A
Acetone	58.08	0.7845	600	40	50
KPi buffer	N/A	N/A	N/A	2450	2500

Waste [from mg] = 2740
E factor = 464

Turner's group work with GOx: Benzyl alcohol to benzoic acid (SI, ref. 7)
14% yield = 0.03915 mg

Table S4: Reaction table for Turner's group work to evaluate waste generated and the E factor.

Reagents	MW [g/mol]	Density [g/mL]	µmol	Mass [mg]	Volume [µL]
Benzyl alcohol	108.1	1.044	2.5	0.27	x
GOx	N/A	N/A	N/A	0.25	x
catalase	N/A	N/A	N/A	0.055	x
KPi buffer	N/A	N/A	N/A	250	250

Waste [from mg] = 250.5
E factor = 6399

Grogan's group work with UPOs: Cinnamyl alcohol to cinnamic acid (SI, ref. 8)
38% yield = 41 mg

Table S5: Reaction table for Grogan's group work to evaluate waste generated and the E factor.

Reagents	MW [g/mol]	density [g/mL]	µmol	Mass [mg]	volume [mL]
Cinnamic alcohol	134.2	1.040	800	107.3	5.179
UPO	N/A	N/A	N/A	240	N/A
H_2O_2	34.01	1.44	1.800	3472	5
KPi buffer	N/A	N/A	N/A	30000	30
H_2O	18.05	1	N/A	18000	18
MeCN	41.05	0.78	N/A	9432	12

Waste [from mg]= 61210
E factor = 1500

8.2 Calculation for NAD^+ cofactor recycling using NOx CFE

To evaluate the sustainability of our methodology from a green chemistry perspective, we compared the energy consumption required for NAD^+ recycling using NOx cell-free extracts (CFEs) versus acetone.

Biotransformation scale
Substrate concentration: 20 mM
Volume: 2.5 mL
Mmol of substrate to be converted: **0.05 mmol $_{sub}$**

Amount of NOx CFE required per 2.5 mL scale biotransformation: 10 mg
Amount of NOx CFE required per mmol of alcohol oxidized (assuming quantitative conversion):
200 mg $_{CFE}$ mmol^{-1} $_{sub}$

*For the production of NOx CFE, 1 g of glucose is used to produce 0.5 g of dry cells.[9] This process requires ca 15 kJ per gram of glucose consumed.[10] Since 400 mg of dry cells are needed to obtain 200 mg of CFE, 800 mg of glucose is needed for the production of 200 mg of CFE. This requires the consumption of **12 KJ of energy**, which is the estimated energy requirement per mmol of alcohol oxidized in the biotransformation.*
*Additional processing steps including cell lysis, centrifugation and lyophilization. To obtain 10 mg of dry lyophilized CFE by processing 100 mg of wet cells suspended in 1 mL buffer with an input of 100 W for 5 mins (10 s ON, 10 s OFF), the sonication consumes 0.300 kJ.[11] Therefore, the production of 200 mg CFE would require **6 KJ for sonication**.*
*The final step is the lyophilization, which requires around 3 kJ to perform the sublimation of 1 g of ice.[12] By processing 100 mg of wet cells to obtain 10 mg of dry NOx CFE, we need to evaporate 80 mg of water (the remaining 10 mg are cell debris). This requires 0.27 kJ only for sublimation. Therefore, the production of 200 mg CFE would theoretically require 5.4 KJ for sublimation. However, during the lyophilisation process, the energy needed for the sublimation represents only the 45%, while the remaining 55% is associated with vacuum pumping, condensation, system heating, and environmental losses.[13] Therefore, the **total energy required for the lyophilization process is 12 KJ** for the production of 200 mg CFE.*

Total energy required to produce NOx required per mmol of alcohol oxidized (assuming quantitative conversion):

(12 KJ cell growing + 6 KJ sonication + 12 KJ lyophilization) $\mathbf{mmol^{-1}}_{\mathbf{sub}}$ = 30 KJ $\mathbf{mmol^{-1}}_{\mathbf{sub}}$

8.3 Calculation for NAD^+ cofactor recycling using acetone

Amount of acetone required per 2.5 mL scale biotransformation: 50 µL
Amount of acetone required per mmol of alcohol oxidized (assuming quantitative conversion):
1 $\mathbf{mL}_{\mathbf{acetone}}$ $\mathbf{mmol^{-1}}_{\mathbf{sub}}$
Considering acetone density, this is equal to:
0.784 $\mathbf{g}_{\mathbf{acetone}}$ $\mathbf{mmol^{-1}}_{\mathbf{sub}}$

Acetone is industrially produced via the cumene process, which involves oxidation of cumene to hydroperoxide, followed its decomposition into acetone and phenol. The energy consumption for this process ranges between 2000 and 3000 MJ per ton of acetone, corresponding to ***2.5 kJ per gram of acetone produced****.* To this amount, we need to add the energy required for distillation, which is **2.35 *kJ per gram of acetone distilled. If we also consider the recycling of the acetone after the reaction—through fractionation from isopropanol and the extraction solvent—this contribution can approximately be counted twice. Overall, the industrial production and recycling of acetone requires 7.2 kJ per gram of acetone.*[14]**

Energy consumption for the production of acetone in our biotransformation: **5.60 kJ $\mathbf{mmol^{-1}}_{\mathbf{sub}}$**

8.4 Calculation for productivity (Space-time-yield)

Taking into consideration the three cases reported in section 8.1, we calculated the productivity of our method and comparing it to the other examples.

1) STY our approach: $\frac{5.4\ mg}{2.5\ mL\ x\ 16\ h} = 0.135\frac{mg}{mL\ h}$

2) STY Turner's paper: $\frac{0.03915\ \text{mg}}{0.250\ mL\ x\ 6\ h} = 0.0261\frac{mg}{mL\ h}$

3) STY Grogan's paper: $\frac{41\ \text{mg}}{65\ mL\ x\ 16\ h} = 0.0394\frac{mg}{mL\ h}$

9. NMR spectra

NMR: 2.5 ppm DMSO, 3.52 ppm Internal standard (Dioxane)

Pentanoic acid. **^{1}H-NMR** (400 MHz, DMSO-d6): δ (ppm) 11.07 (s, 1H), 2.35 (t, J = 7.5 Hz, 2H), 1.72 – 1.58 (m, 2H), 1.39 – 1.27 (m, 2H), 0.99 – 0.85 (m, 3H).

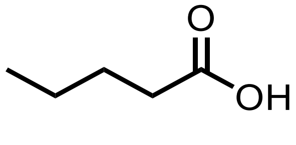

Hexanoic acid. **^{1}H-NMR** (400 MHz, DMSO-d6): δ (ppm) 11.03 (s, 1H), 2.34 (t, J = 7.5 Hz, 2H), 1.70 – 1.57 (m, 2H), 1.39 – 1.25 (m, 4H), 0.96 – 0.84 (m, 3H).

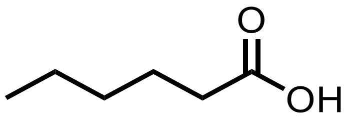

Heptanoic acid. **^{1}H-NMR** (400 MHz, DMSO-d6): δ (ppm) 10.99 (s, 1H), 2.23 (t, J=7.4, 1H), 1.50-1.38 (m, 1H), 1.26-1.17 (m, 4H), 0.81 (t, J=7.0, 1H).

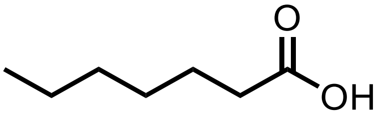

Octanoic acid. **^{1}H-NMR** (400 MHz, DMSO-d6): δ (ppm) 10.96 (s, 1H), 2.34 (t, J = 7.5 Hz, 2H), 1.67 – 1.59 (m, 2H), 1.37 – 1.23 (m, 8H), 0.92 – 0.84 (m, 3H).

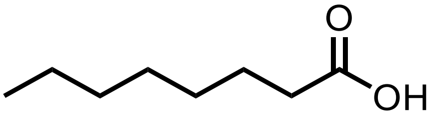

Decanoic acid. **^{1}H-NMR** (400 MHz, DMSO-d6): δ (ppm) 11.95 (s, 1H), 2.18 (t, J = 7.4 Hz, 2H), 1.48 (t, J = 7.1 Hz, 2H), 1.20–1.30 (m, 12H), 0.85 (t, J = 6.7 Hz, 3H).

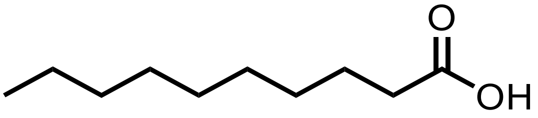

2-Methyl pentanoic acid. **^{1}H-NMR** (400 MHz, DMSO-d6): δ (ppm) 11.95 (s, 1H), 2.37 – 2.21 (m, 1H), 1.62 – 1.42 (m, 1H), 1.38 – 1.16 (m, 3H), 1.03 (d, J = 7.0 Hz, 3H), 0.85 (t, J = 7.2 Hz, 3H).

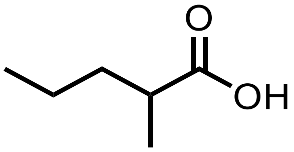

3-Methyl pentanoic acid. **^{1}H-NMR** (400 MHz, DMSO-d6): δ (ppm) 11.64 (s, 1H), 2,35 (dd, J = 6.0 Hz, 1H), 2.14 (dd, J = 8.0 Hz, 1H), 1.83−1.95 (m, 1H), 1.34−1.45 (m, 1H), 1.19−1.30 (m, 1H), 0.96 (d, J = 6.7 Hz, 3H), 0.90 (t, J = 7.4 Hz, 3H).

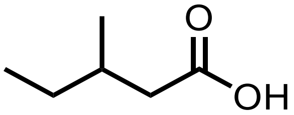

4-Methyl pentanoic acid. **^{1}H-NMR** (400 MHz, DMSO-d6): δ (ppm) 11.85 (s, 1H), 2.44 – 2.19 (m, 2H), 1.58 (ddd, J = 13.7, 12.7, 6.4 Hz, 1H), 1.55 – 1.50 (m, 2H), 0.90 (d, J = 6.5 Hz, 6H).

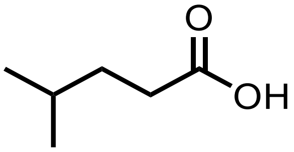

Benzoic acid. **^{1}H NMR** (400 MHz, DMSO-d6): δ (ppm) 12.95 (s, 1H), 7.97 (d, J = 8.3 Hz, 2H), 7.58 (t, J = 7.4 Hz, 1H), 7.47 (t, J = 8.2 Hz, 2H).

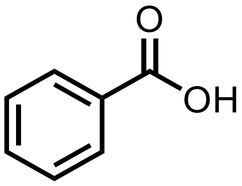

4-Chlorobenzoic acid. **^{1}H NMR** (400 MHz, DMSO-d6): δ (ppm) 13.13 (s, 1H), 7.92 (d, J = 8.6 Hz, 2H), 7.50 (d, J = 8.5 Hz, 2H).

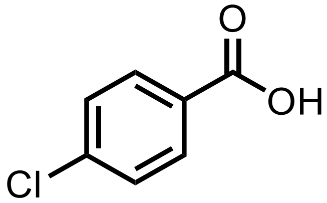

3-Chlorobenzoic acid. **^{1}H NMR** (400 MHz, DMSO-d6): δ (ppm) 13.27 (s, 1H), 7.86-7.88 (m, 2H), 7.64-7.61 (m, 1H), 7.48 (t, J = 7.8 Hz, 1H).

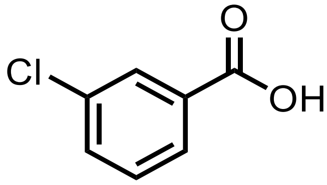

2-Chlorobenzaldehyde. **^{1}H NMR** (400 MHz, DMSO-d6): δ (ppm) 13.35 (s, 1H), 7.80 – 7.77 (m, 1H), 7.52 – 7.47 (m, 2H), 7.40-7.36 (m, 1H).

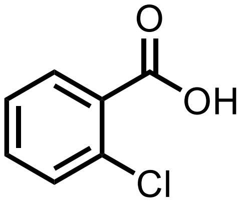

4-Methoxybenzoic acid. **^{1}H NMR** (400 MHz, DMSO-d6): δ (ppm) 12.60 (s, 1H), 7.90 (d, J = 8.9 Hz, 2H), 6.98 (d, J = 8.9 Hz, 2H), 3.79 (s, 3H).

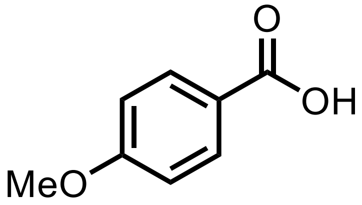

4-Fluorobenzoic acid. **^{1}H NMR** (400 MHz, DMSO- d6): δ (ppm) 13.01 (s, 1H), 7.99 (dd, J = 8.9, 5.6 Hz, 2H), 7.27 (t, J = 8.9 Hz, 2H).

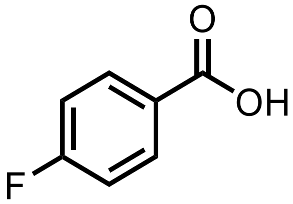

4-Methylbenzoic acid: **^{1}H NMR**(400 MHz, DMSO- d6): δ (ppm) 12.77 (s, 1H), 7.84 (d, J = 8.2 Hz, 2H), 7.25 (d, J = 8.1 Hz, 2H), 2.32 (s, 3H).

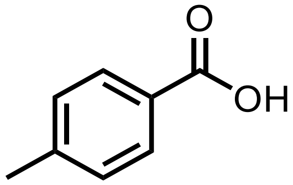

3-Methylbenzoic acid. **^{1}H NMR** (400 MHz, DMSO-d6): δ (ppm) 12.85 (s, 1H), 7.76 (s, 1H), 7.75 (d, J = 7.2 Hz, 1H), 7.39 (d, J = 7.6 Hz, 1 H), 7.36 (t, J = 7.6 Hz, 1H), 2.32 (s, 3H).

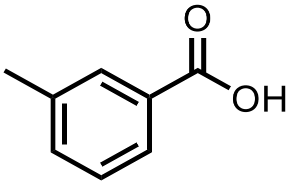

2-Methylbenzoic acid. **^{1}H NMR** (400 MHz, DMSO-d6): δ (ppm) 12.79 (s, 1H), 7.83 (d, J = 8.1 Hz, 1H), 7.45-7.41 (m, 1H), 7.27 (t, J = 8.4 Hz, 2H), 2.52 (s, 3H).

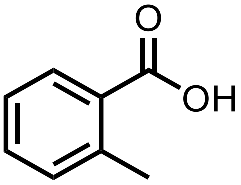

Cinnamic acid. **^{1}H NMR** (400 MHz, DMSO-d6): δ (ppm) 12.30 (s, 1H), 7.69 (m, 2H), 7.60 (d, J-=16.09Hz, 1H), 7.42 (m, 3H), 6.53 (d, J-=16.09Hz, 1H).

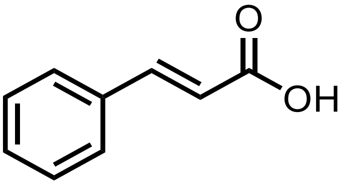

2-Phenylacetic acid. **^{1}H-NMR** (400 MHz, DMSO-d6): δ (ppm) 11.37 (s, 1H), 7.40 – 7.28 (m, 5H), 3.67 (s, 2H).

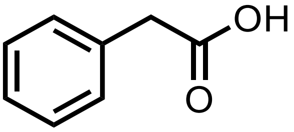

3-Phenylpropanoic acid. **^{1}H-NMR** (400 MHz, DMSO-d6): δ (ppm) 12.16 (s, 1H), 7.19–7.29 (m, 5H), 2.83 (t, J = 7.6 Hz, 2H), 2.54 (t,J = 7.7 Hz, 2H).

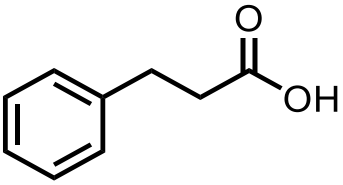

4-Phenylbutanoic acid. **^{1}H-NMR** (400 MHz, DMSO-d6): δ (ppm) 12.06 (s, 1H), 7.31 - 7.26 (m, 2H), 7.20 - 7.15 (m, 3H), 2.58 (dd, J = 8.6, 6.7 Hz, 2H), 2.21 (t, J = 7.4 Hz, 2H), 1.88 – 1.69 (m, 2H).

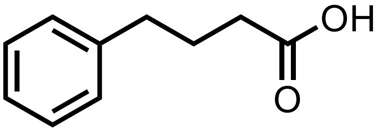

2-Phenylpropanoic acid. **^{1}H NMR** (400 MHz, DMSO-d6): δ (ppm) 12.24 (s, 1H), 7.25-7.39 (m, 5H), 3.76 (q, J = 7.2 Hz, 1H), 1.54 (d, J = 7.2 Hz, 3H)

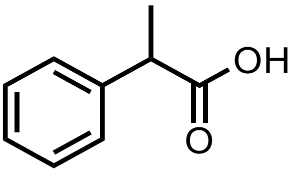

2-Naphthoic acid. **^{1}H NMR** (400 MHz, DMSO-d6): δ (ppm) 13.03 (s, 1H), 8.61 (s, 1H), 8.11 (d, J = 8.1 Hz, 1 H), 7.98–8.00 (m, 3 H), 7.66 (t, J = 6.8 Hz, 1 H), 7.61 (t, J = 7.0 Hz, 1 H).

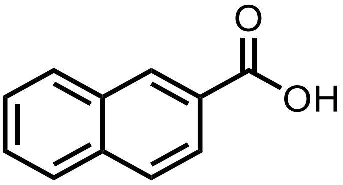

1-Naphthoic acid. **^{1}H NMR** (400 MHz, DMSO-d6): δ (ppm) 13.13 (s, 1H), 8.89 (d, J = 8.5 Hz, 1H), 8.20−8.08 (m, 2H), 8.00 (dd, J = 8.0, 1.6 Hz, 1H), 7.77−7.48 (m, 3H).

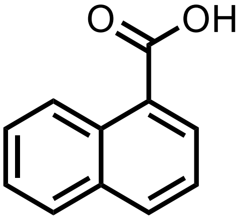

1-Methyl-1H-indole-2-carboxylic acid. **^{1}H NMR** (400 MHz, DMSO-d6): δ (ppm) 12.96 (s, 1H), 7.66 (d, J = 8.0 Hz,1H), 7.54 (dd, J = 8.4, 0.4 Hz, 1H), 7.34−7.29 (m, 1H), 7.23 (d, J =0.8 Hz, 1H), 7.13−7.09 (m, 1H), 4.02 (s, 3H).

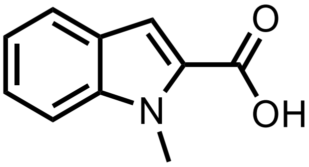

Quinoline-3-carboxylic Acid. **^{1}H NMR** (400 MHz, DMSO-d6): δ (ppm) 13.10 (s, 1H), 9.32 (d, J = 2.1Hz, 1H), 8.94 (d, J = 2.1 Hz, 1H), 8.18 (dd, J = 8.3, 1.5 Hz,1H), 8.09 (d, J = 8.4 Hz, 1H), 7.88 (ddd, J = 8.4, 6.8, 1.5 Hz,1H), 7.69 (ddd, J = 8.1, 6.8, 1.2 Hz, 1H).

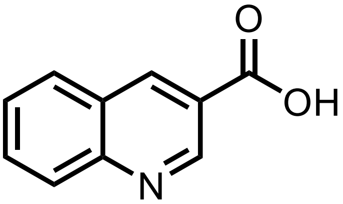

Quinoline-2-carboxylic Acid. **^{1}H NMR** (400 MHz, DMSO-d6): δ (ppm) 13.03 (s, 1H), 8.70 (d, J = 8.5Hz, 1H), 8.33−8.04 (m, 3H), 7.92 (t, J = 7.7 Hz, 1H), 7.78 (t,J = 7.5 Hz, 1H)

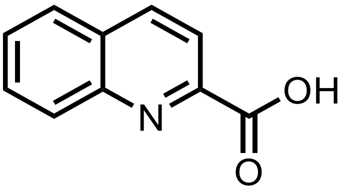

5-(Hydroxymethyl)-2-francarboxylic acid. **^{1}H NMR** (400 MHz, DMSO-d6): δ (ppm) 12.95 (br, 1H), 7.14 (d, J = 3.4 Hz, 1H), 6.46 (d, J = 3.4 Hz, 1H), 5.44 (br, 1H), 4.44 (s, 2H);

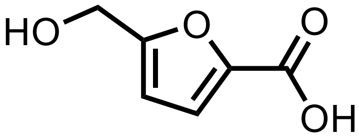

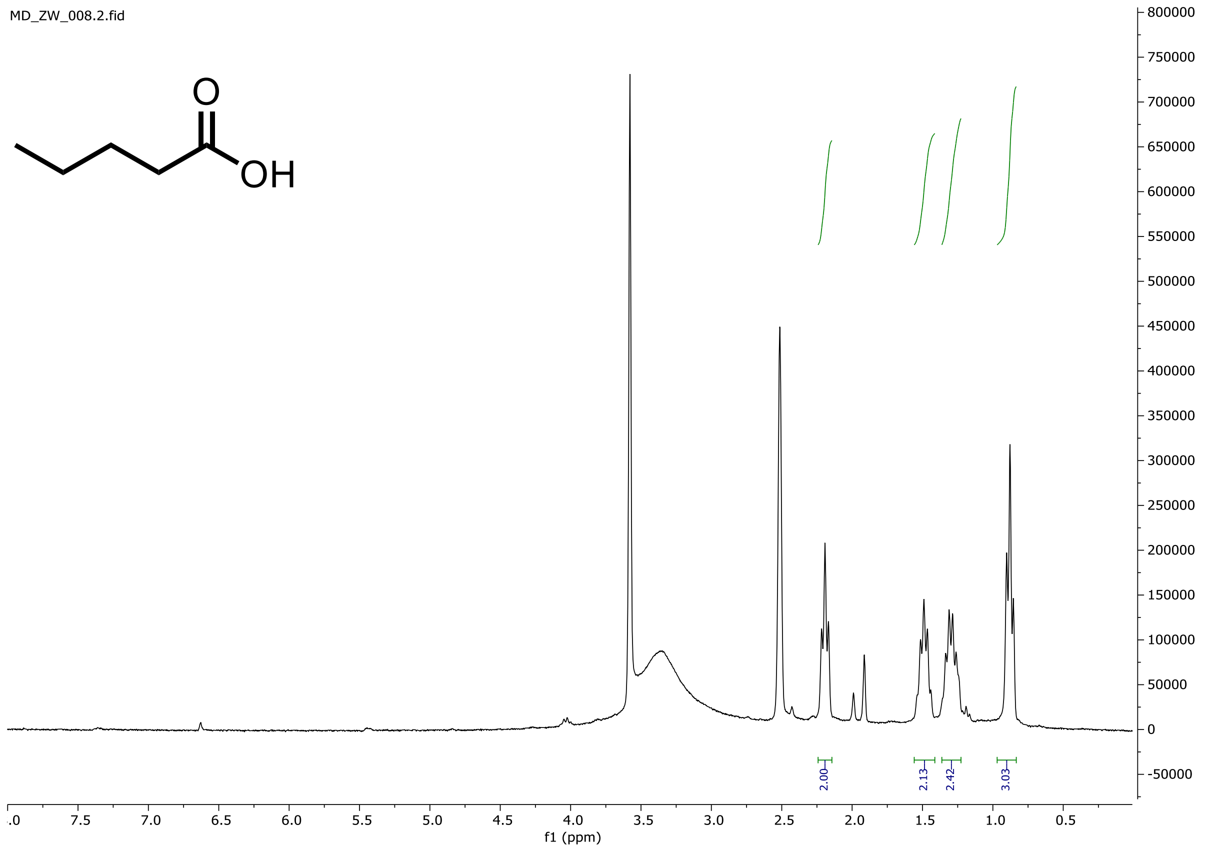
MD_ZW_008.2.fid
O
OH
2.00
2.13
2.42
3.03
f1 (ppm)

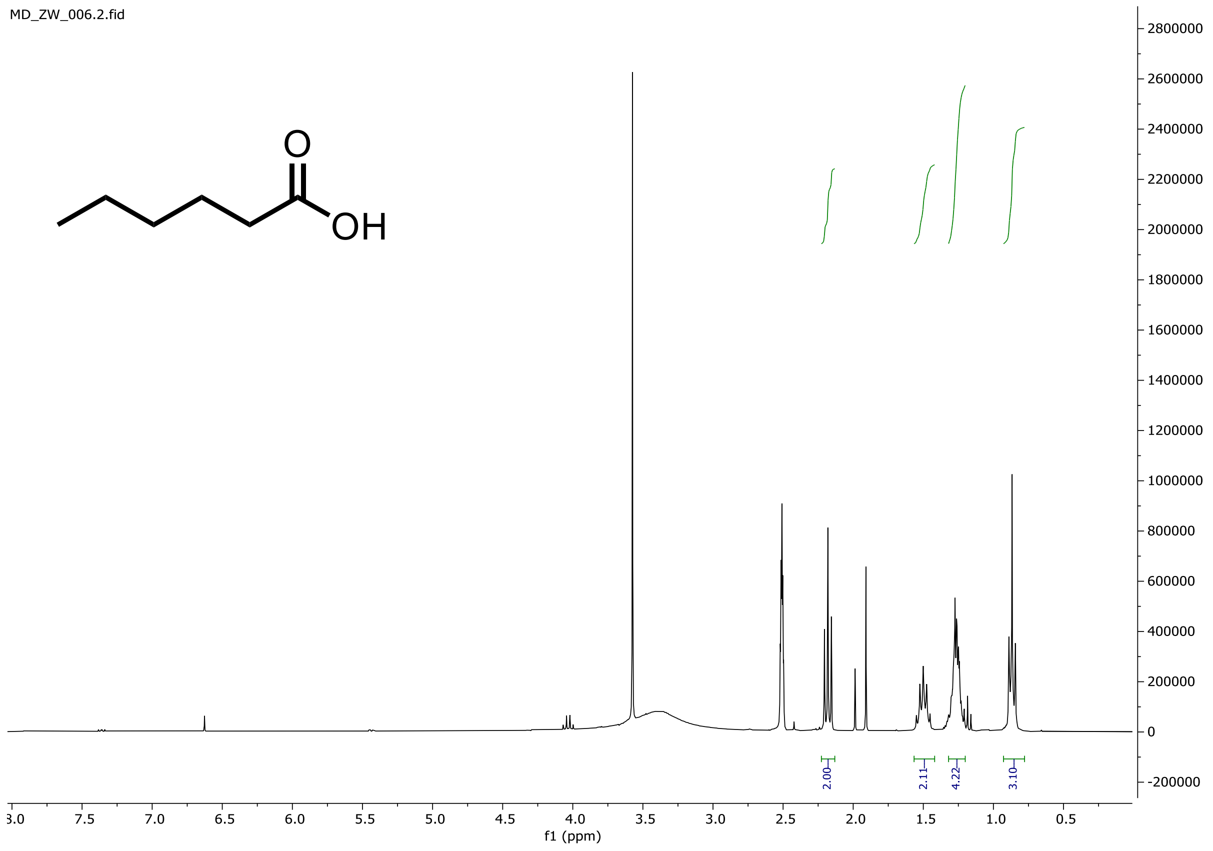
MD_ZW_006.2.fid
O
OH
2.00
2.11
4.22
3.10
f1 (ppm)

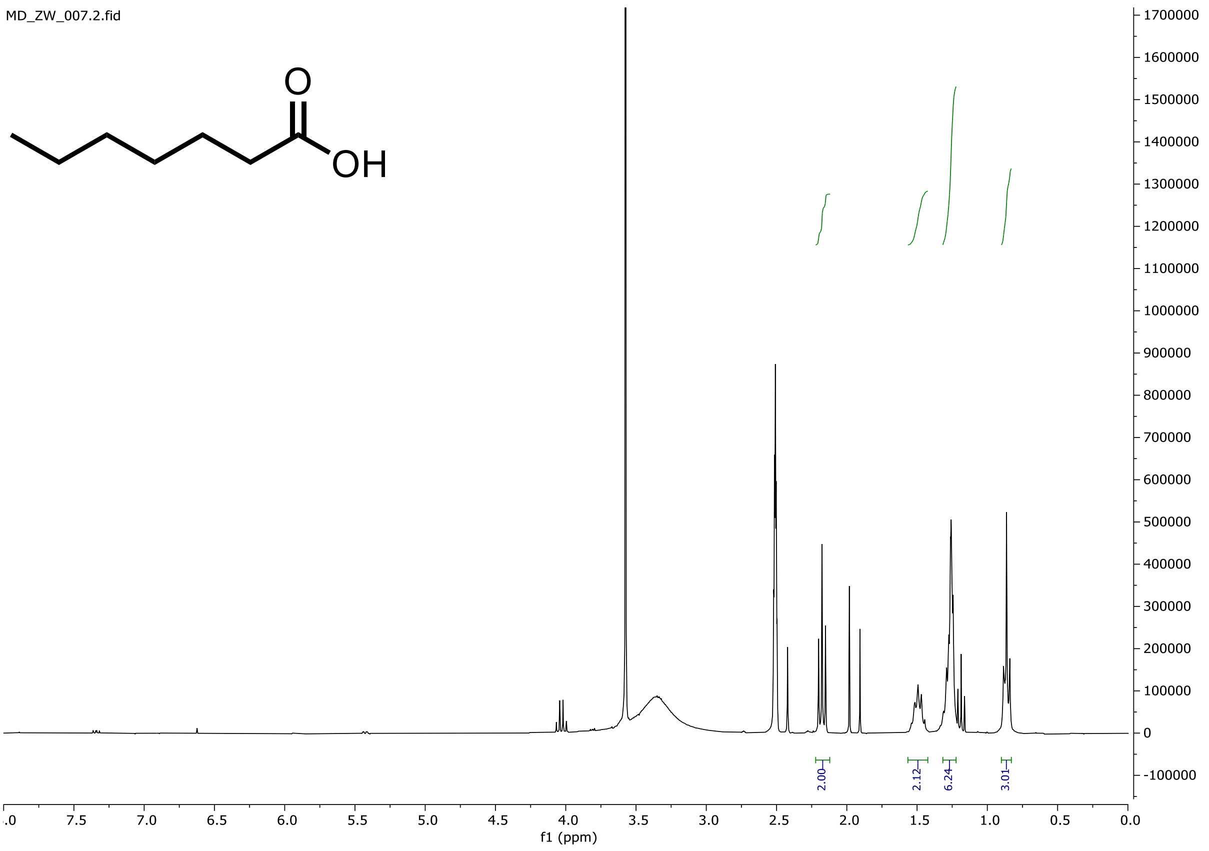
MD_ZW_007.2.fid
O
OH
2.00
2.12
6.24
3.01
f1 (ppm)

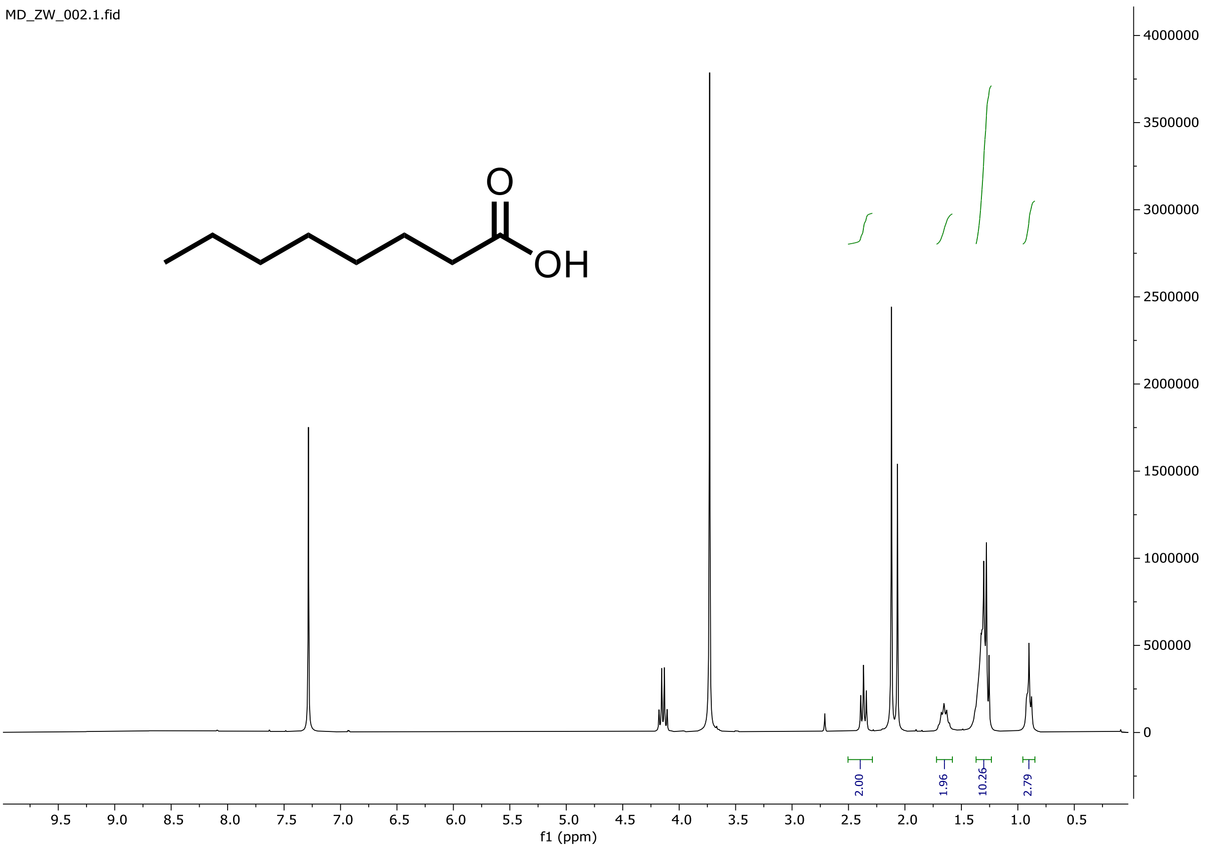
MD_ZW_002.1.fid
O
OH
2.00
1.96
10.26
2.79
f1 (ppm)

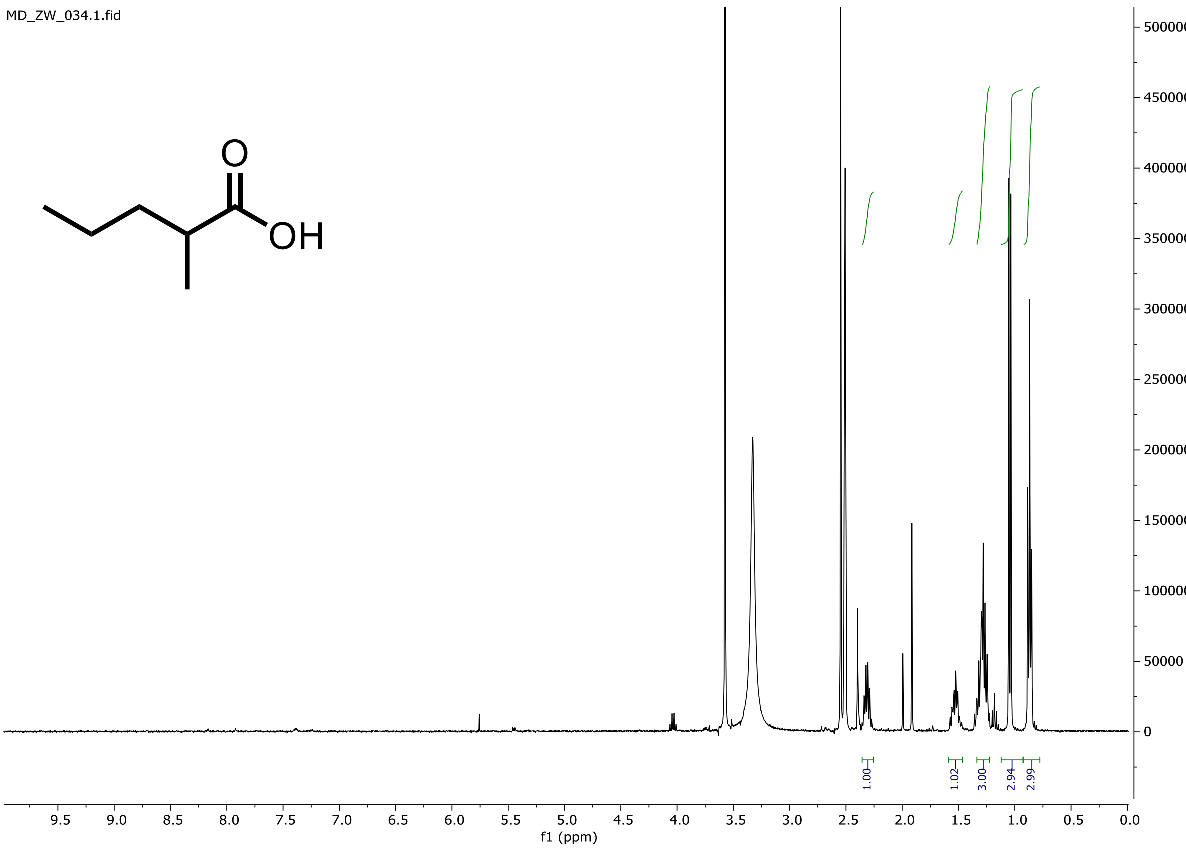
MD_ZW_034.1.fid
O
OH
1.00
1.02
3.00
2.94
2.99
f1 (ppm)

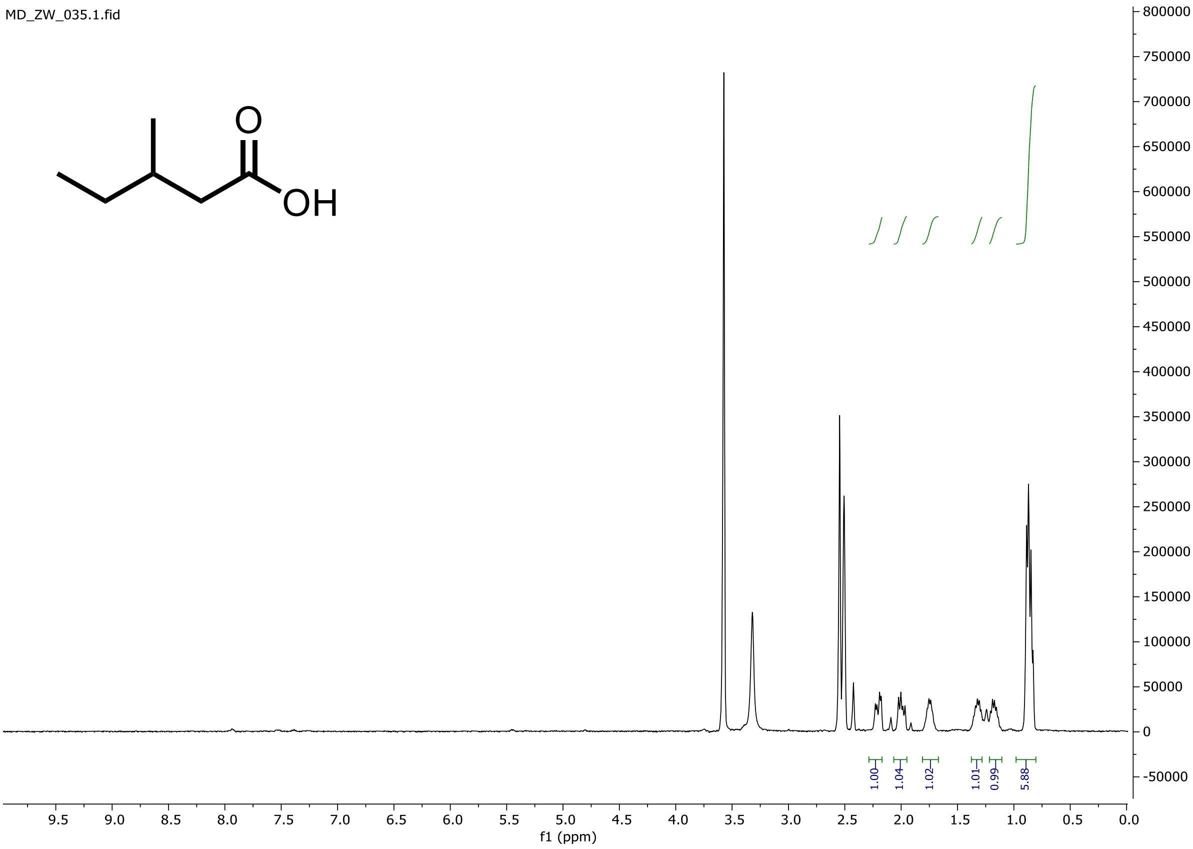
MD_ZW_035.1.fid
O
OH
1.00
1.04
1.02
1.01
0.99
5.88
f1 (ppm)

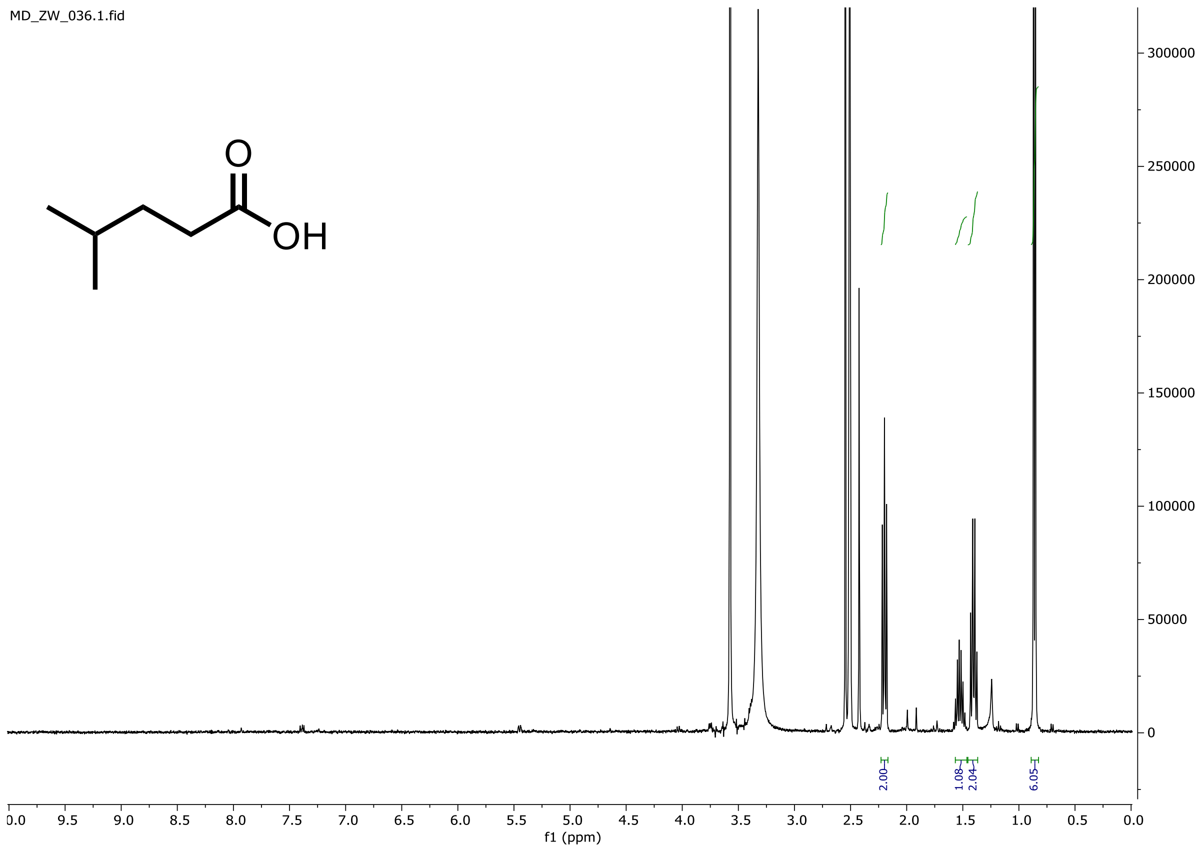
MD_ZW_036.1.fid
O
OH
2.00
1.08
2.04
6.05
f1 (ppm)

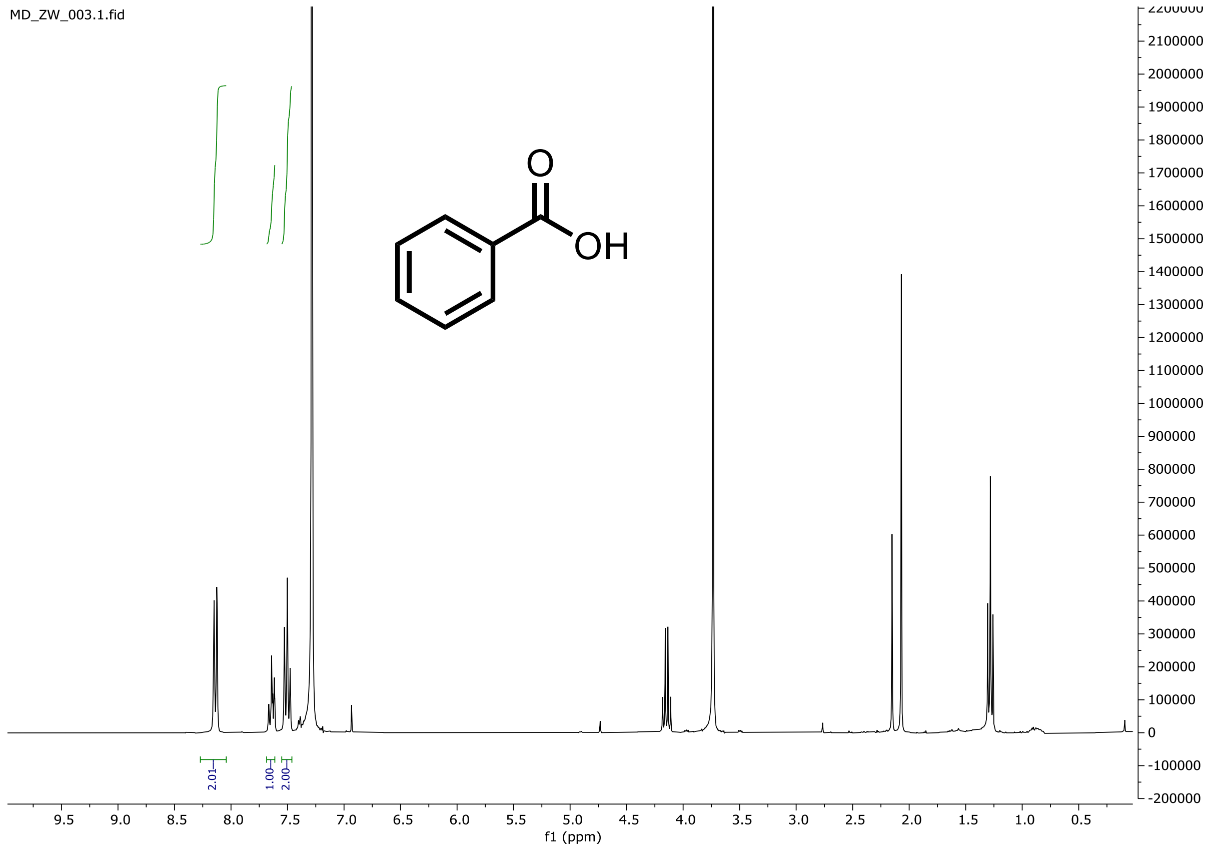
MD_ZW_003.1.fid
O
OH
2.01
1.00
2.00
f1 (ppm)

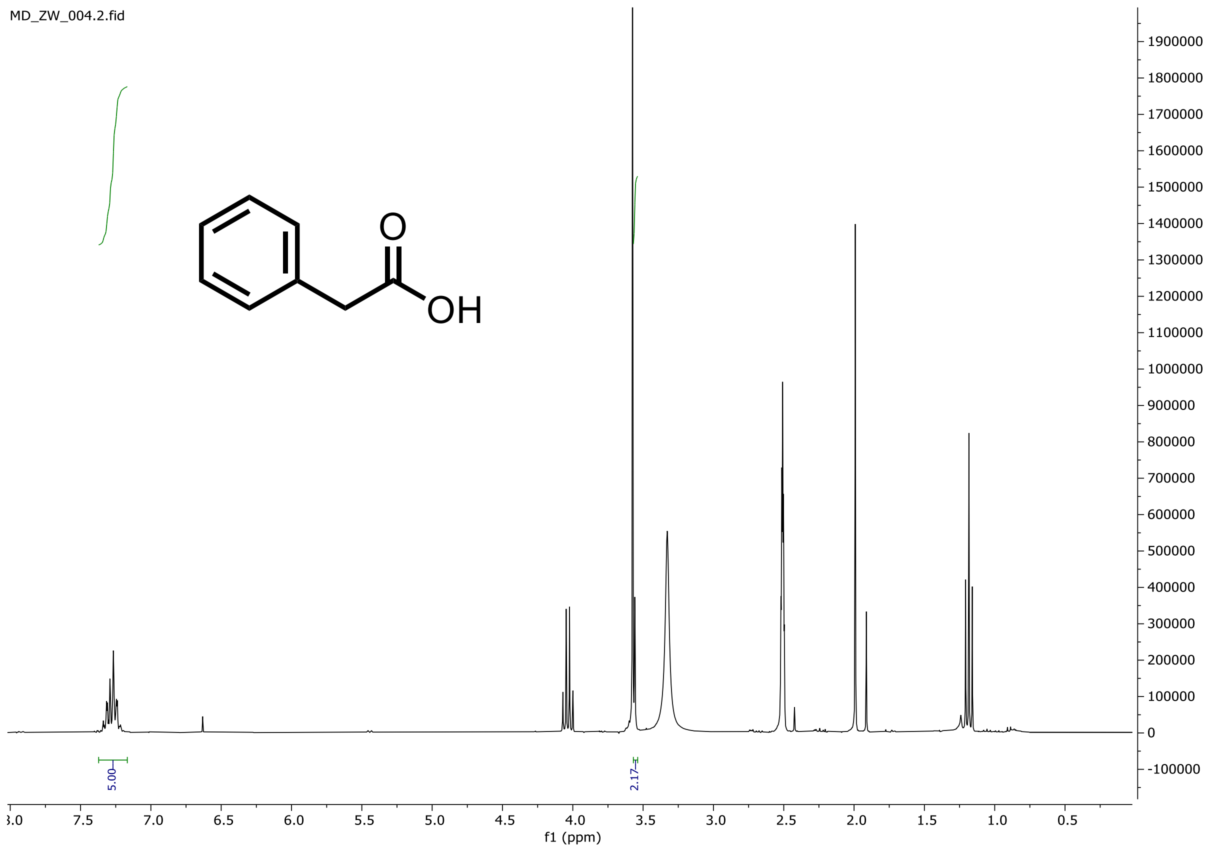
MD_ZW_004.2.fid
OH
O
5.00
2.17
f1 (ppm)

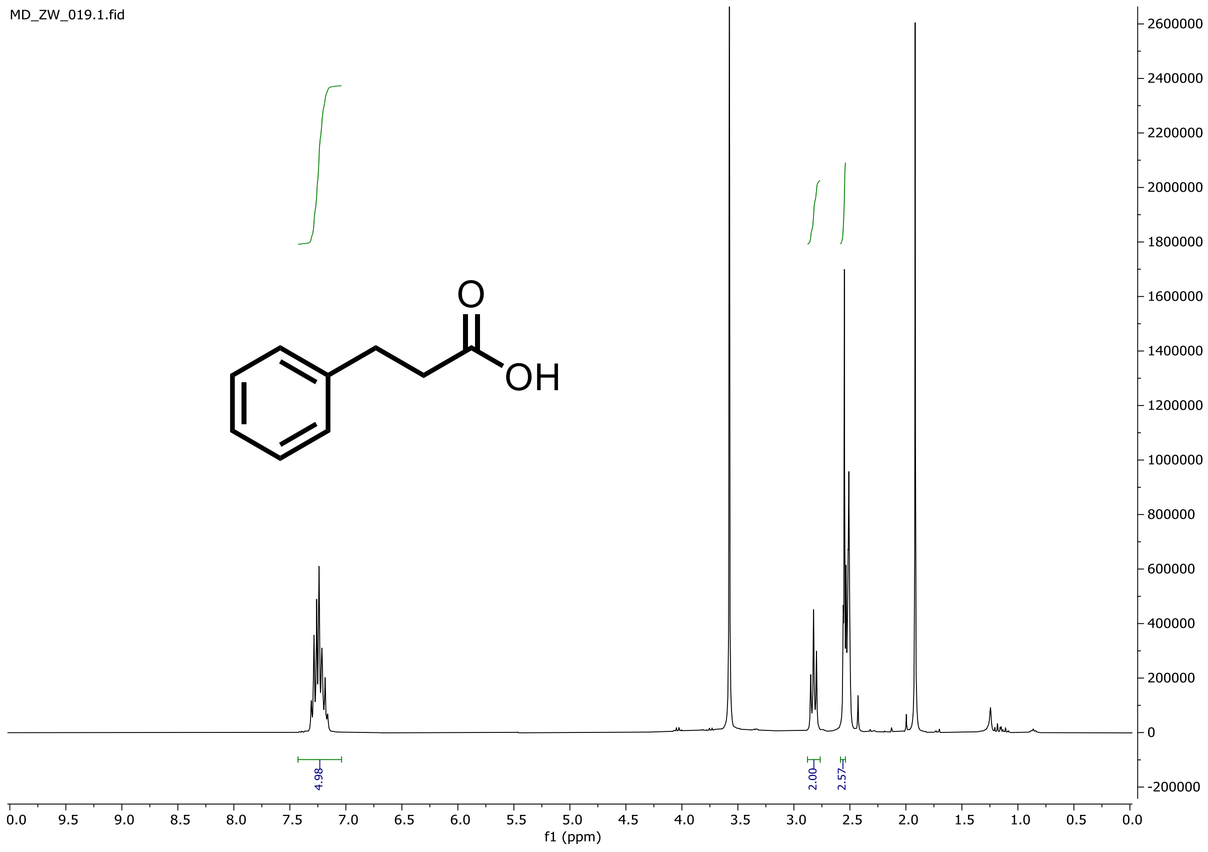
MD_ZW_019.1.fid
OH
O
4.98
2.00
2.57
f1 (ppm)

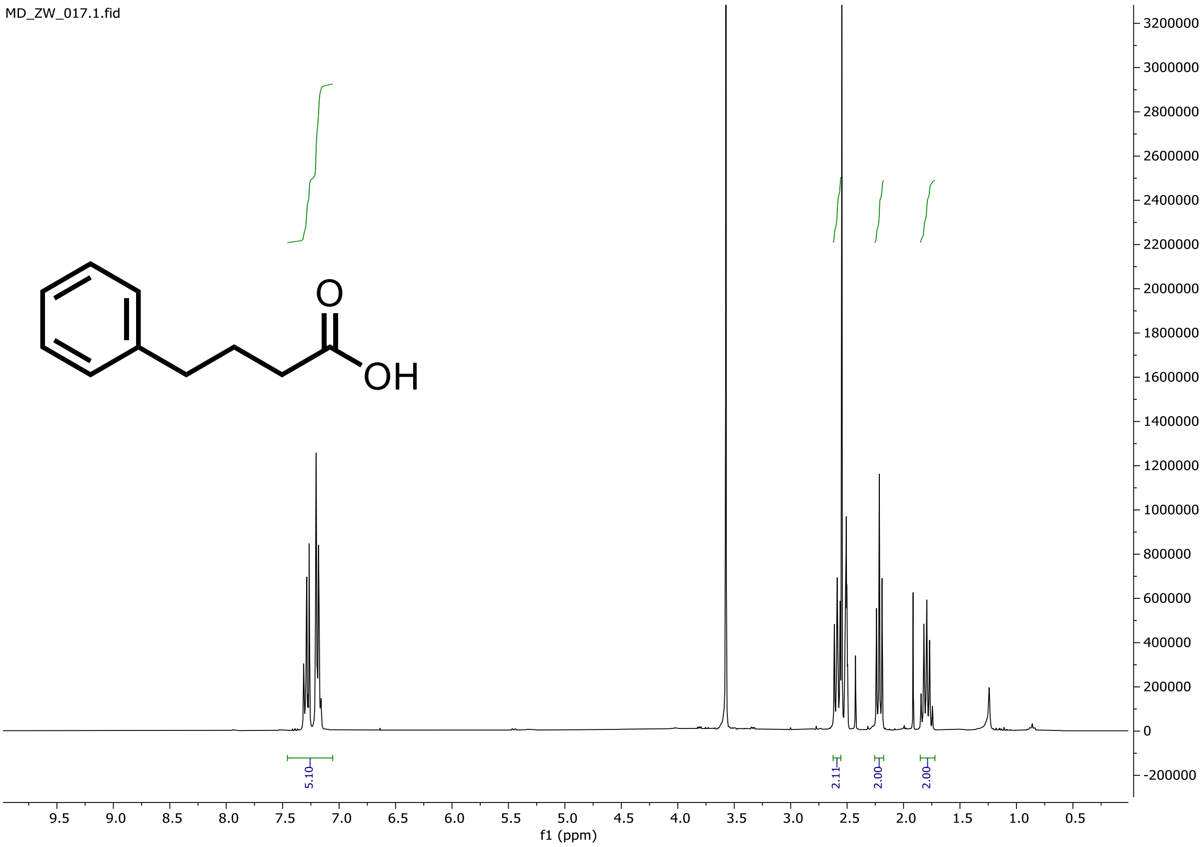
MD_ZW_017.1.fid
O
OH
5.10
2.11
2.00
2.00
f1 (ppm)

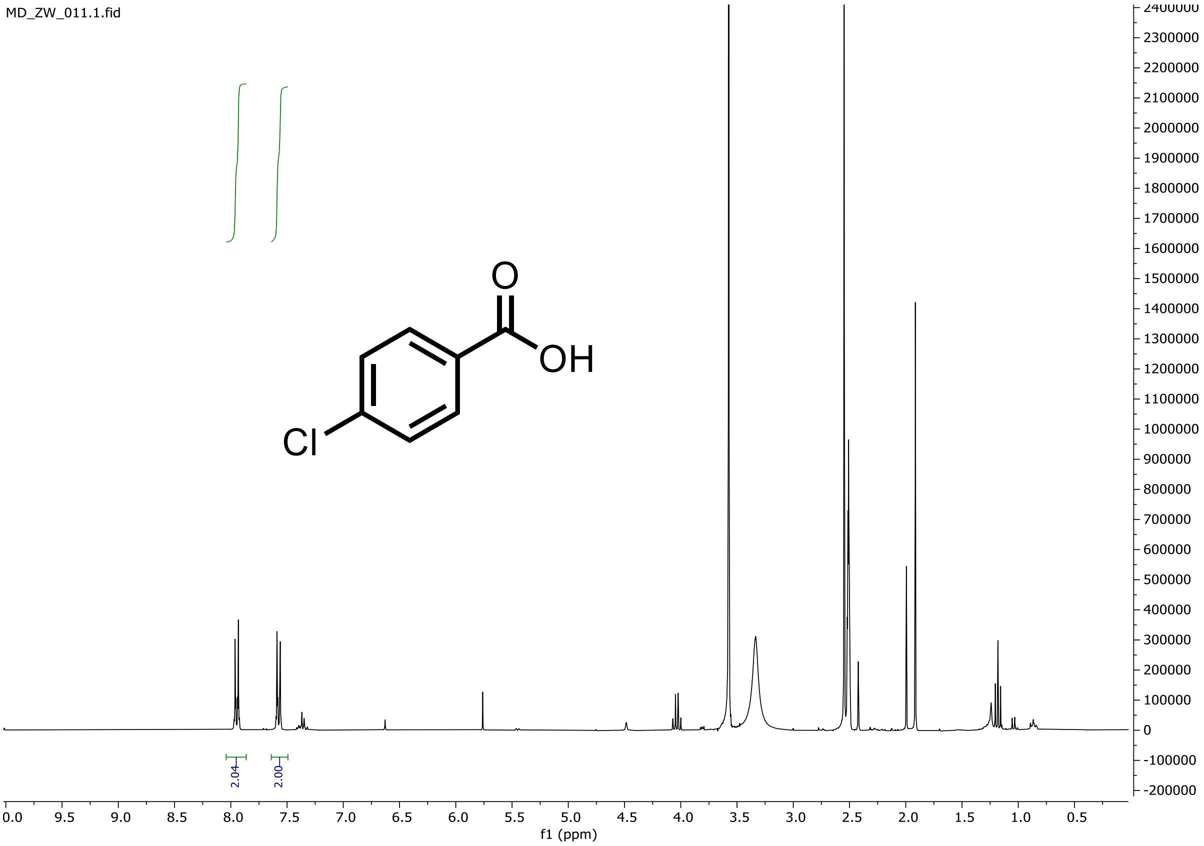
MD_ZW_011.1.fid
O
OH
Cl
2.04
2.00
f1 (ppm)

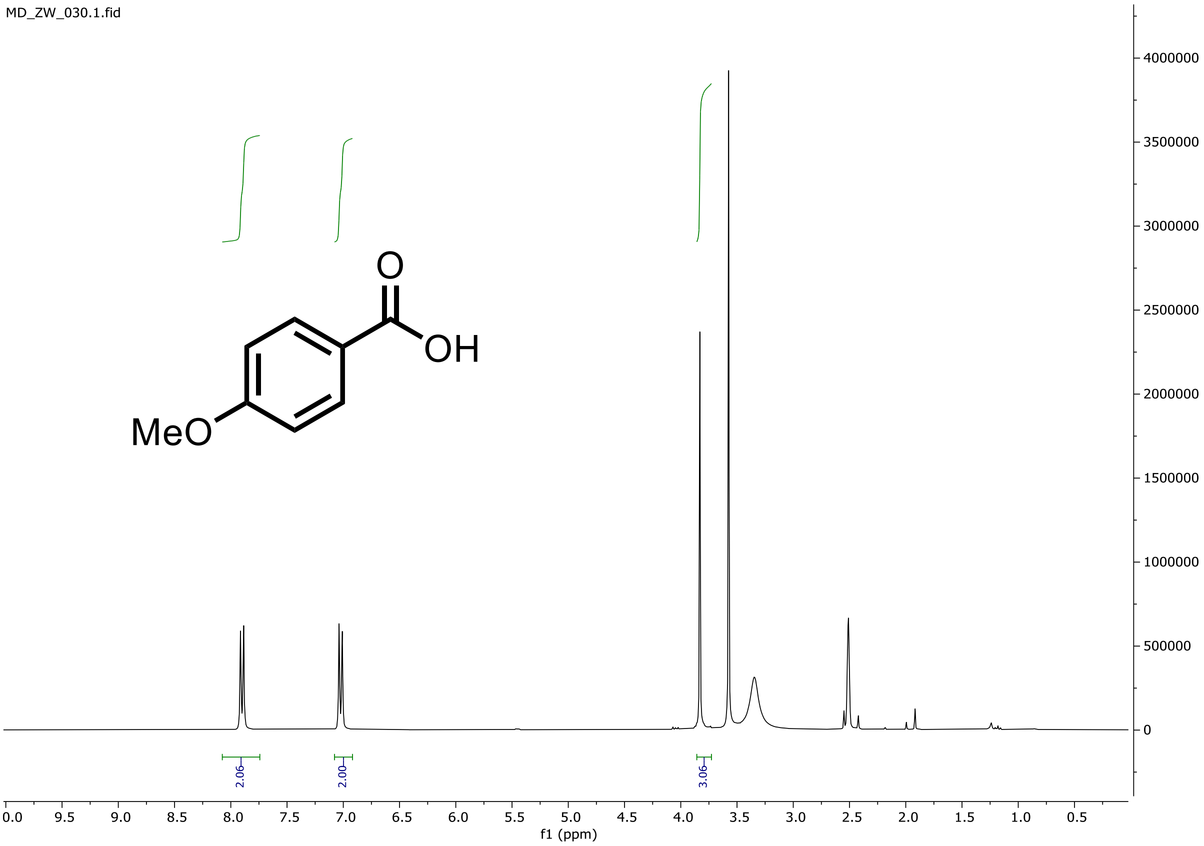
MD_ZW_030.1.fid
O
OH
MeO
2.06
2.00
3.06
f1 (ppm)

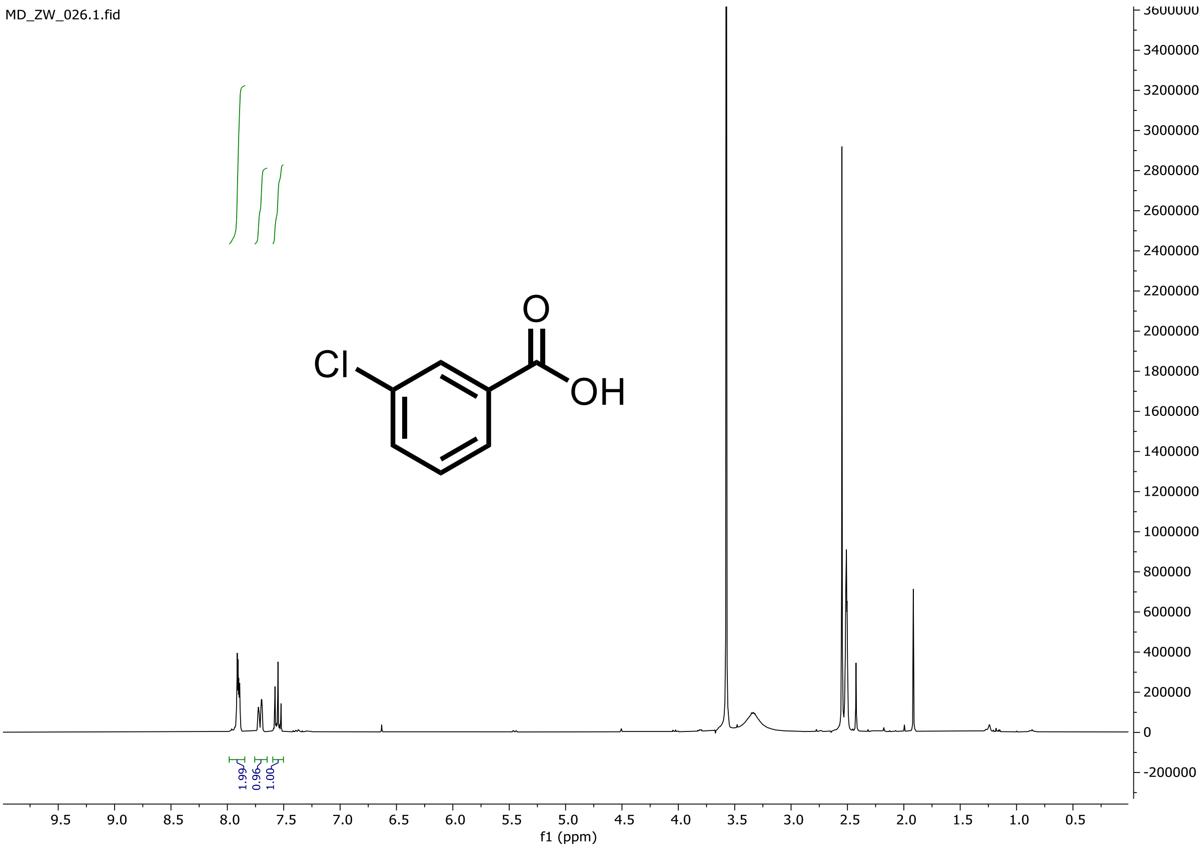
MD_ZW_026.1.fid
O
Cl
OH
1.99
0.96
1.00
f1 (ppm)

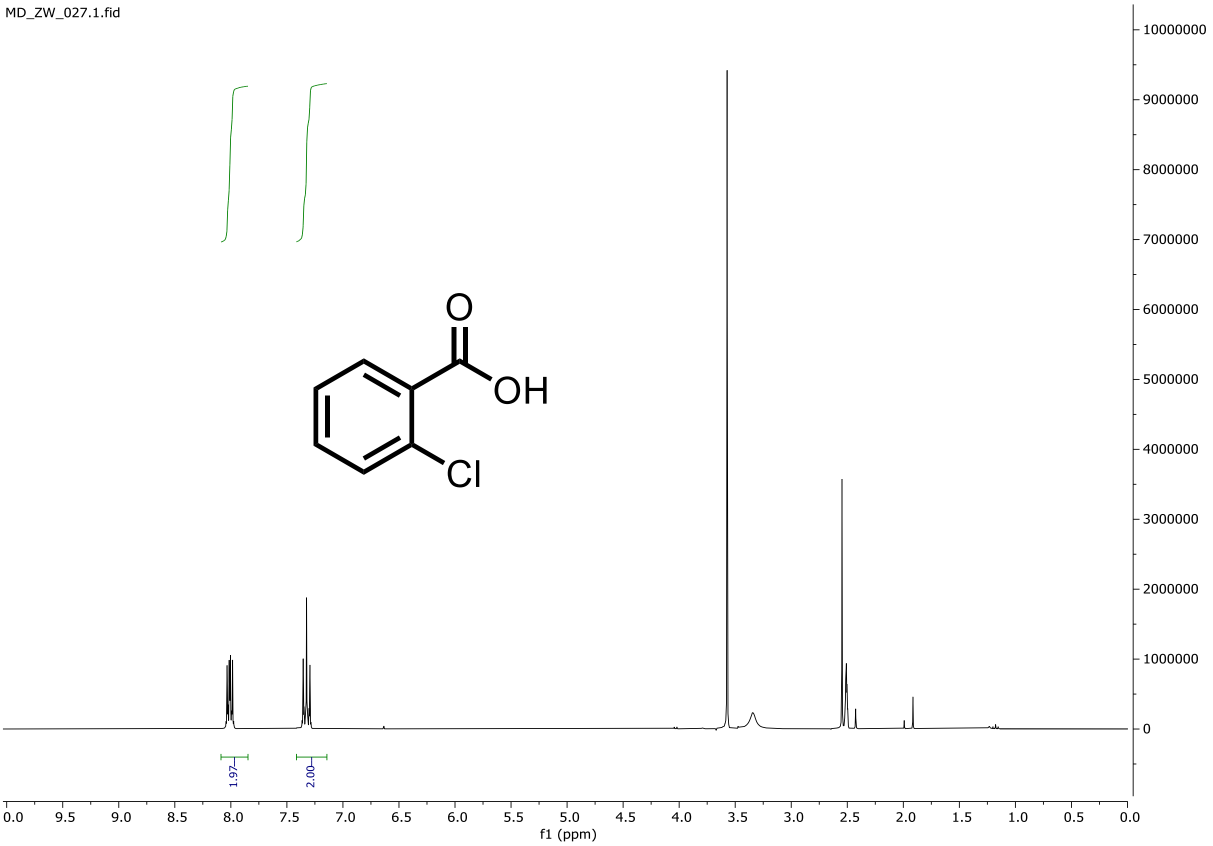
MD_ZW_027.1.fid
O
OH
Cl
1.97
2.00
f1 (ppm)

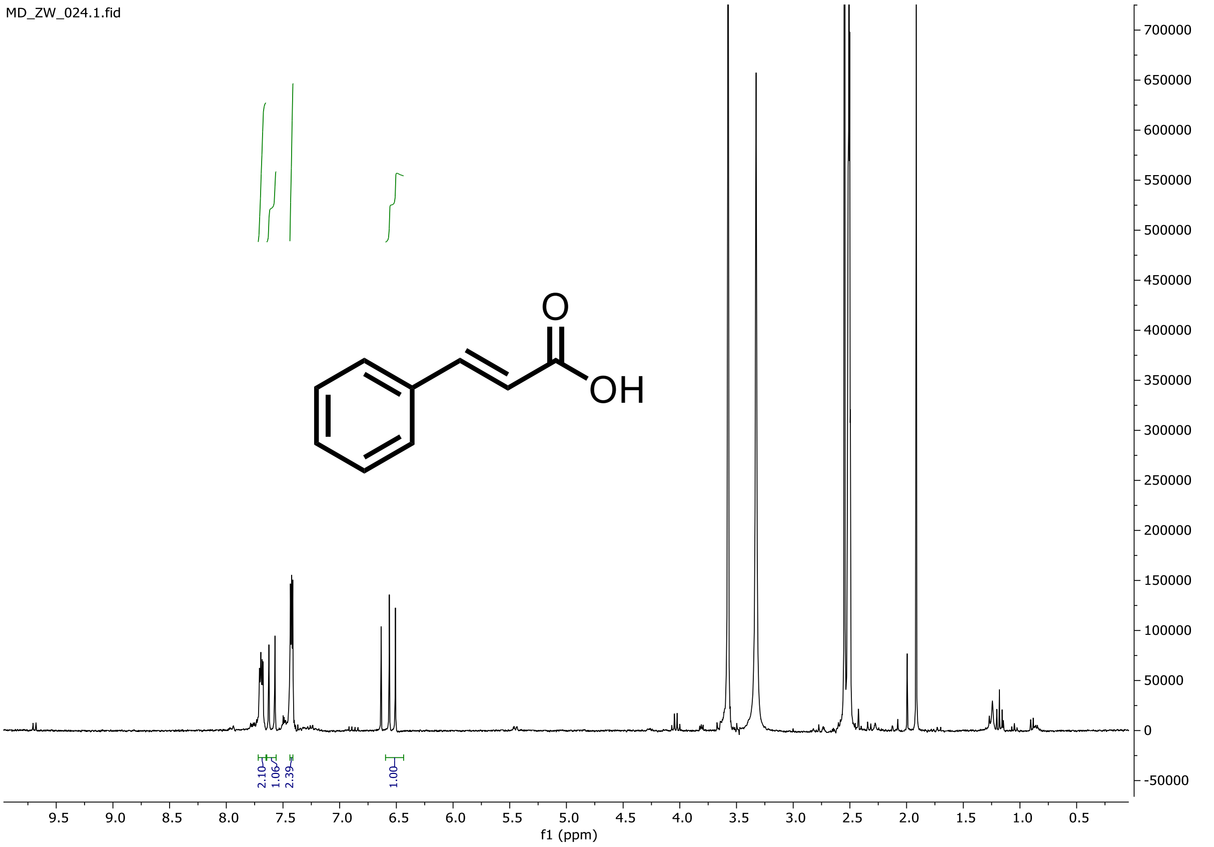
MD_ZW_024.1.fid
O
OH
2.10
1.06
2.39
1.00
f1 (ppm)

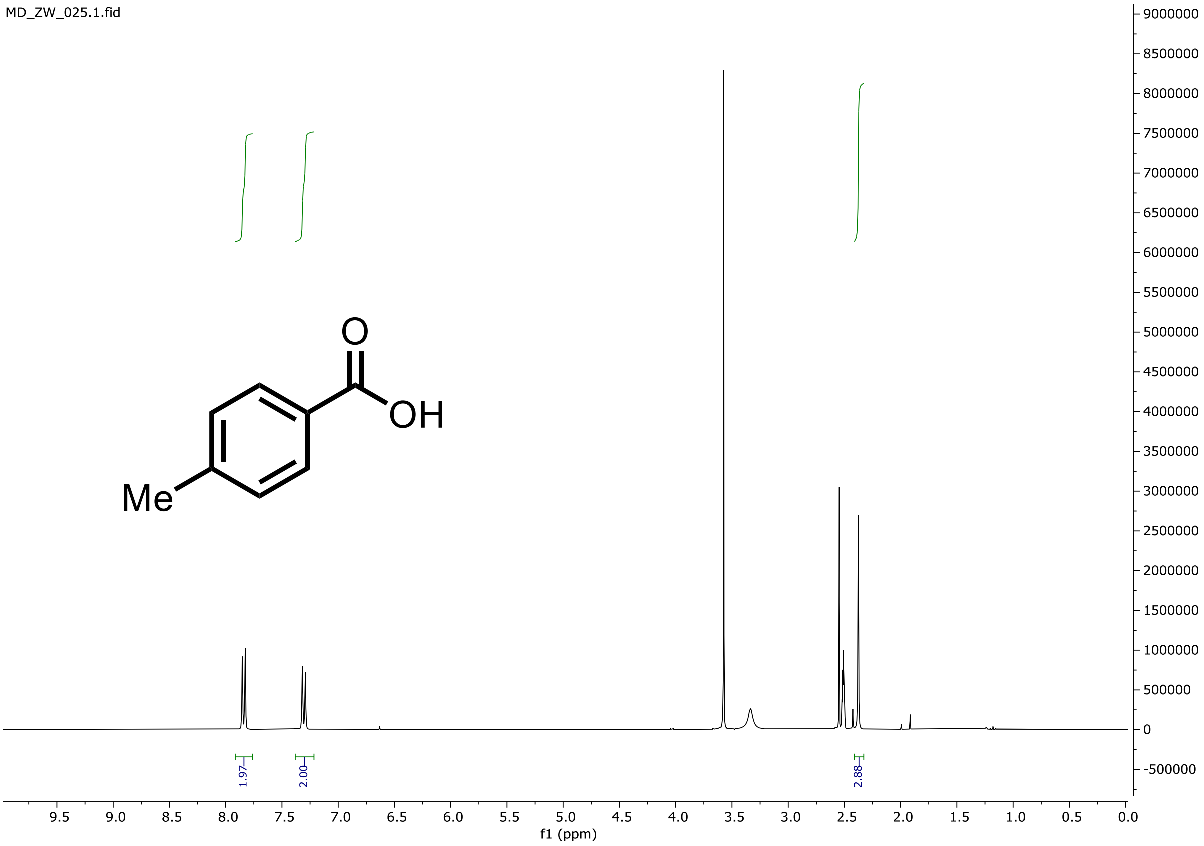
MD_ZW_025.1.fid
O
OH
Me
1.97
2.00
2.88
9.5 9.0 8.5 8.0 7.5 7.0 6.5 6.0 5.5 5.0 4.5 4.0 3.5 3.0 2.5 2.0 1.5 1.0 0.5 0.0
f1 (ppm)
9000000 8500000 8000000 7500000 7000000 6500000 6000000 5500000 5000000 4500000 4000000 3500000 3000000 2500000 2000000 1500000 1000000 500000 0 -500000

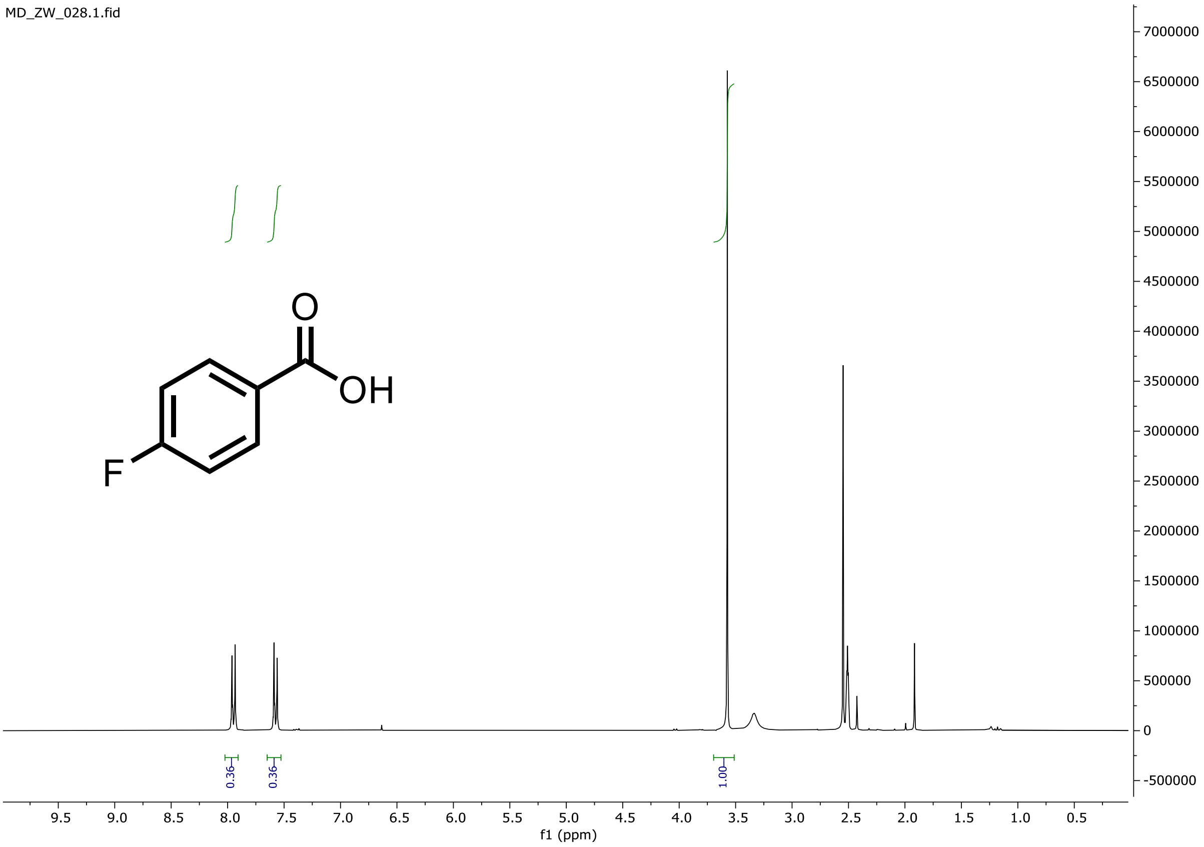
MD_ZW_028.1.fid
O
OH
F
0.36
0.36
1.00
9.5 9.0 8.5 8.0 7.5 7.0 6.5 6.0 5.5 5.0 4.5 4.0 3.5 3.0 2.5 2.0 1.5 1.0 0.5
f1 (ppm)
7000000 6500000 6000000 5500000 5000000 4500000 4000000 3500000 3000000 2500000 2000000 1500000 1000000 500000 0 -500000

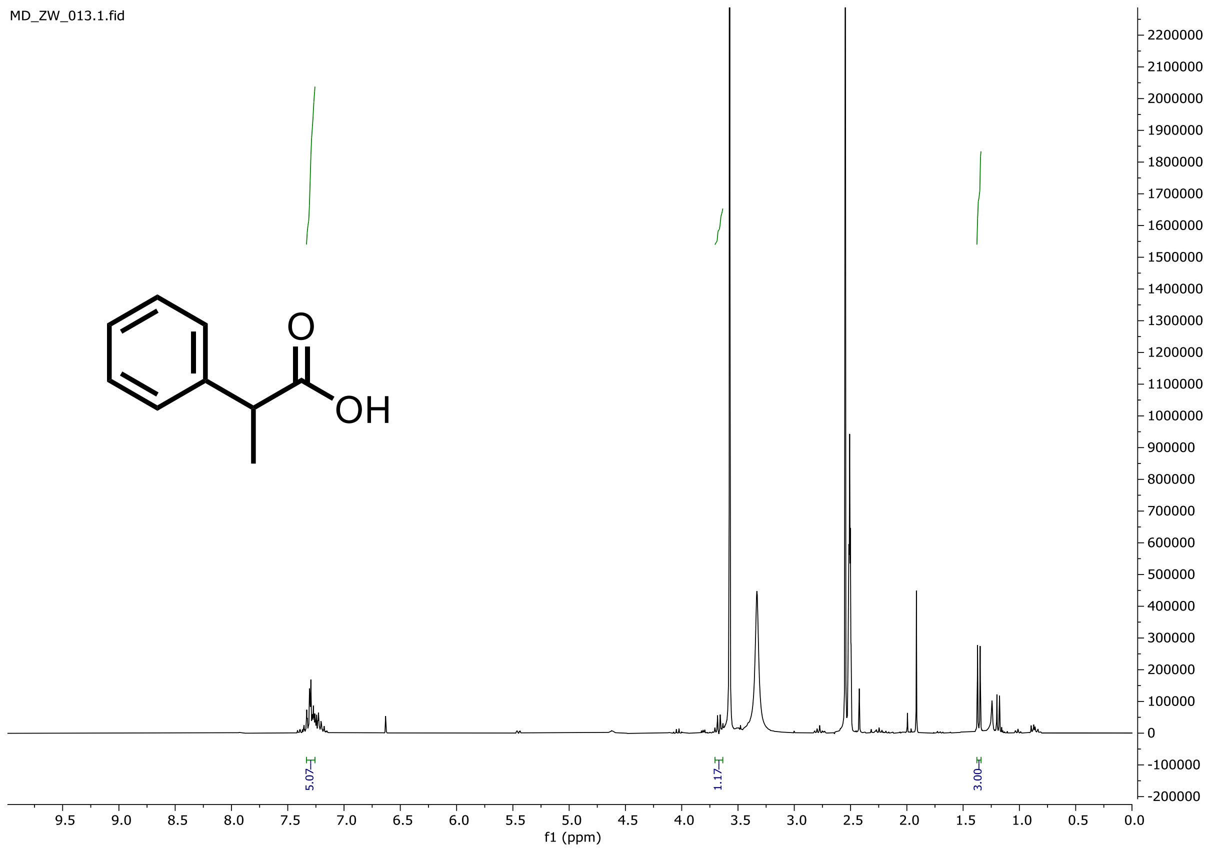
MD_ZW_013.1.fid
OH
O
5.07
1.17
3.00
f1 (ppm)

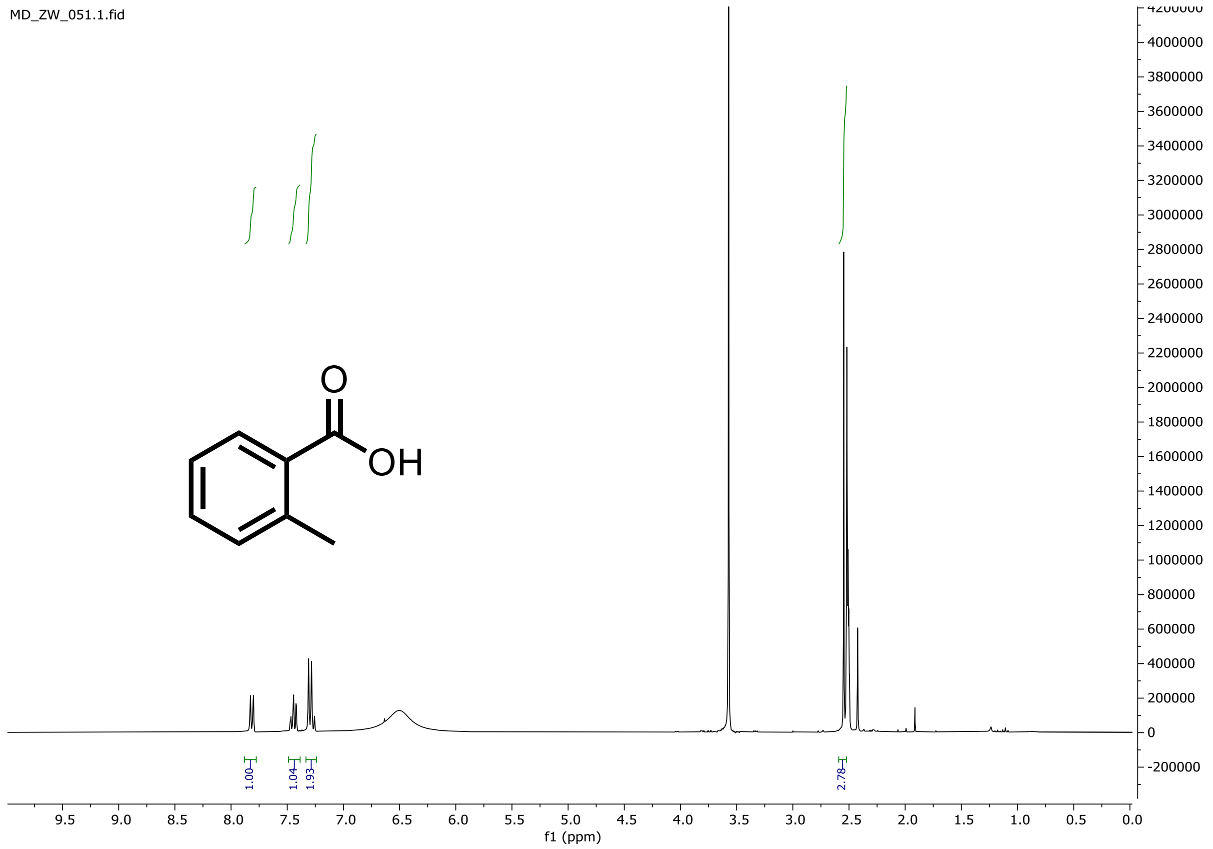
MD_ZW_051.1.fid
O
OH
1.00
1.04
1.93
2.78
f1 (ppm)

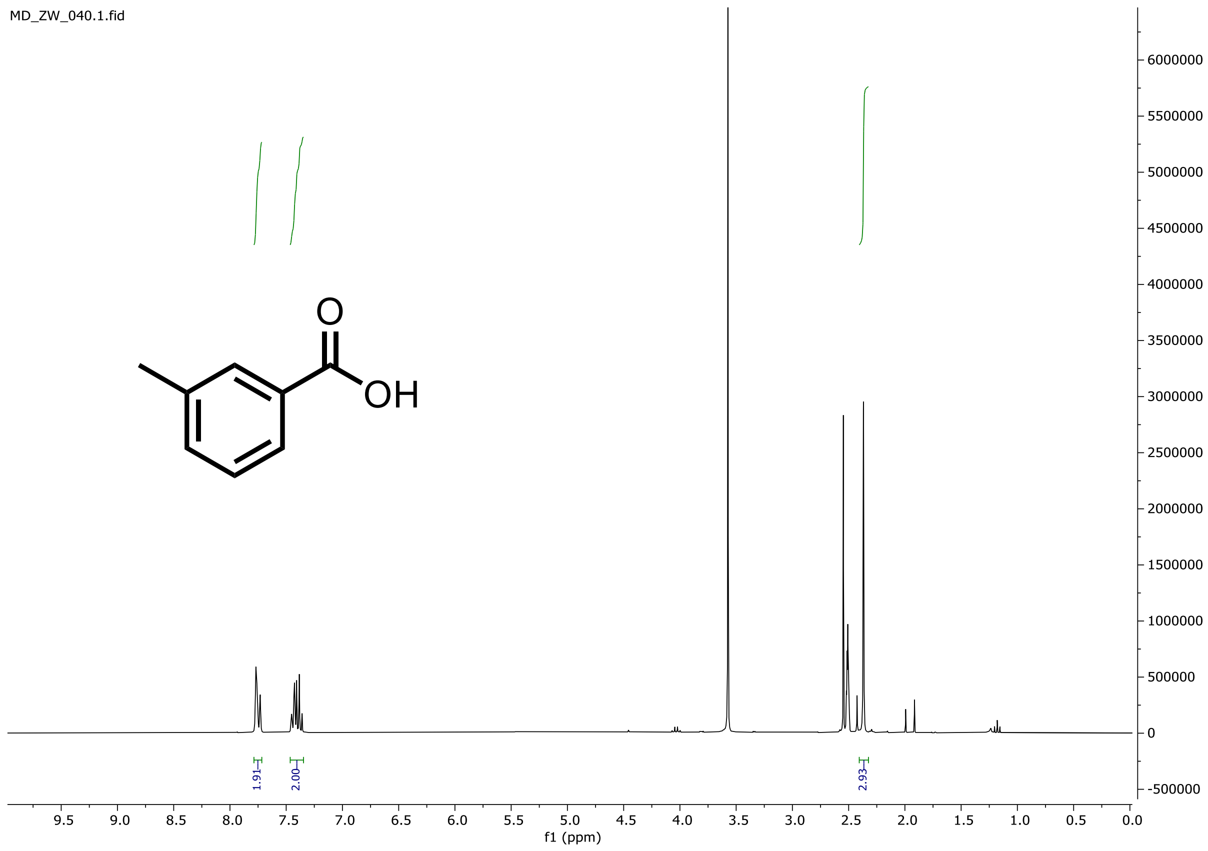
MD_ZW_040.1.fid
O
OH
1.91
2.00
2.93
f1 (ppm)

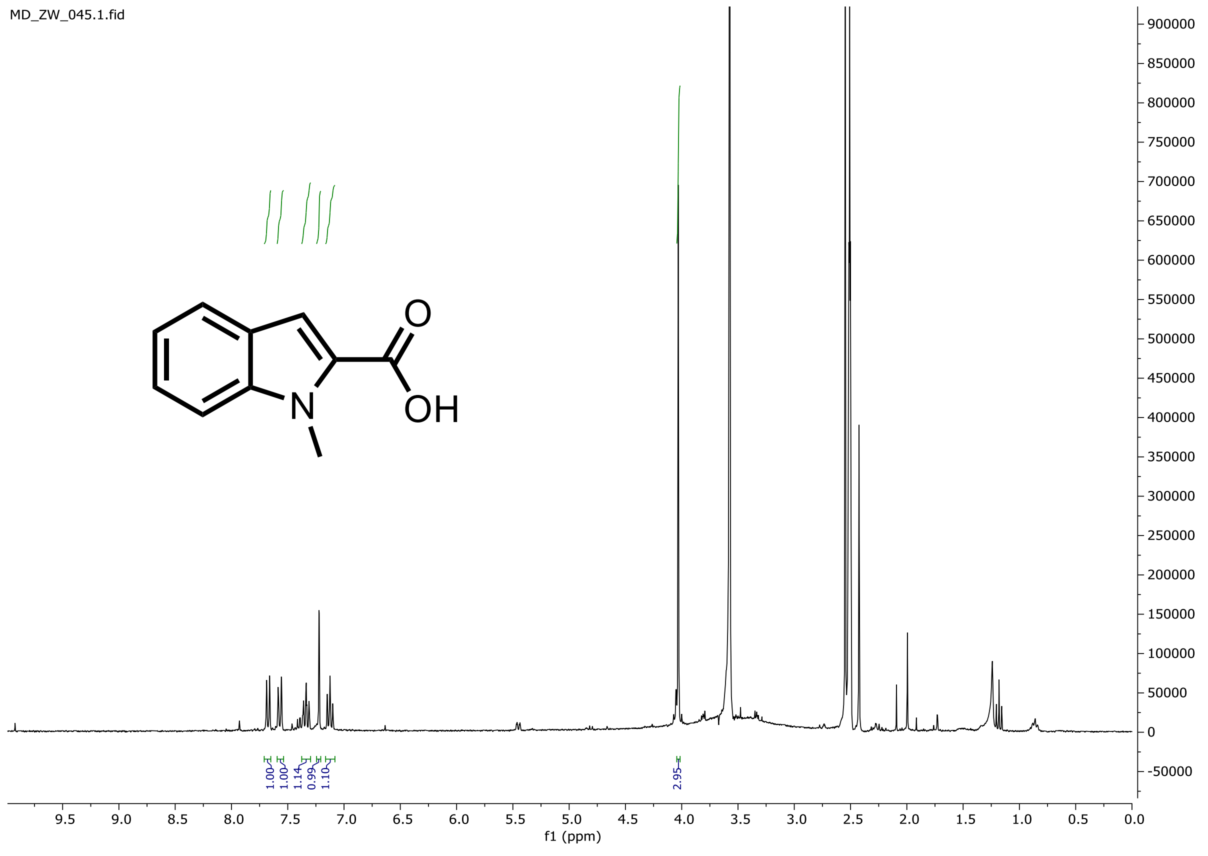
MD_ZW_045.1.fid
O
N
OH
1.00
1.00
1.14
0.99
1.10
2.95
f1 (ppm)

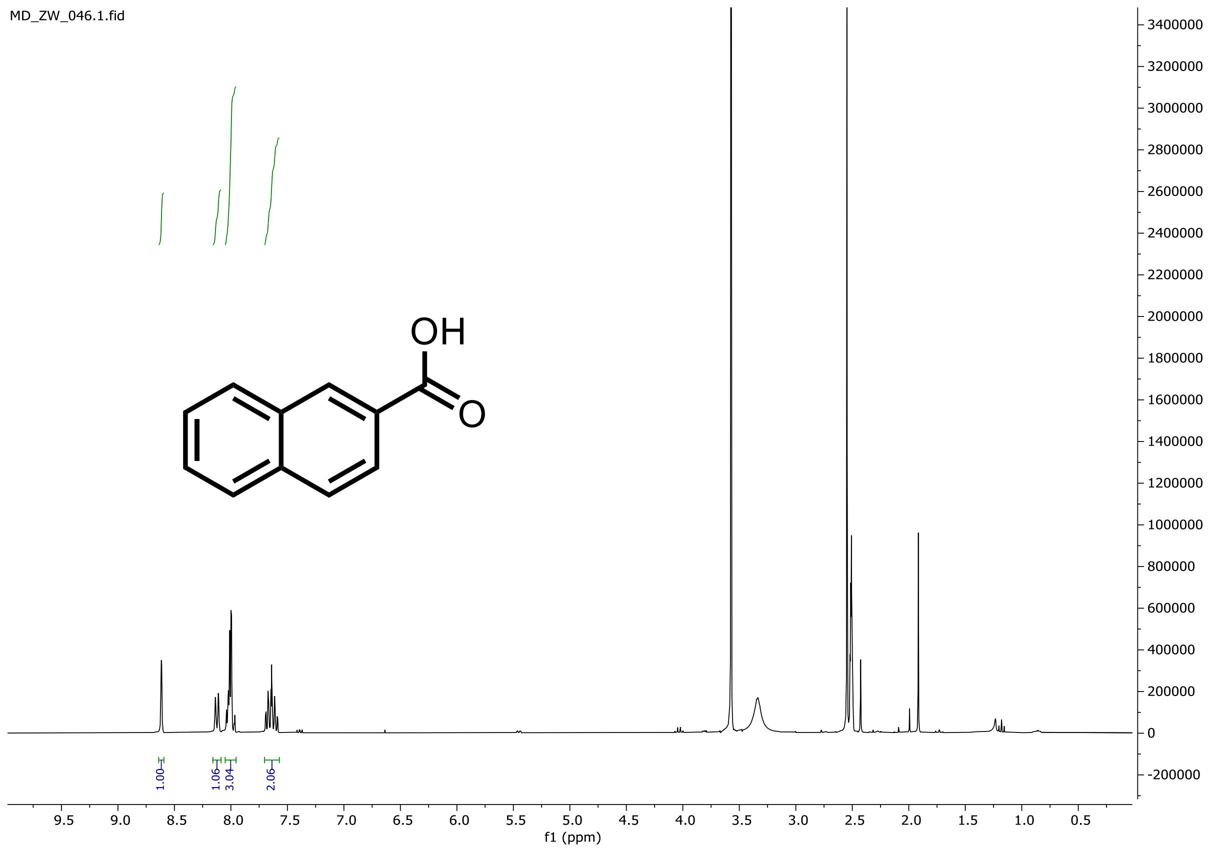
MD_ZW_046.1.fid
OH
O
f1 (ppm)

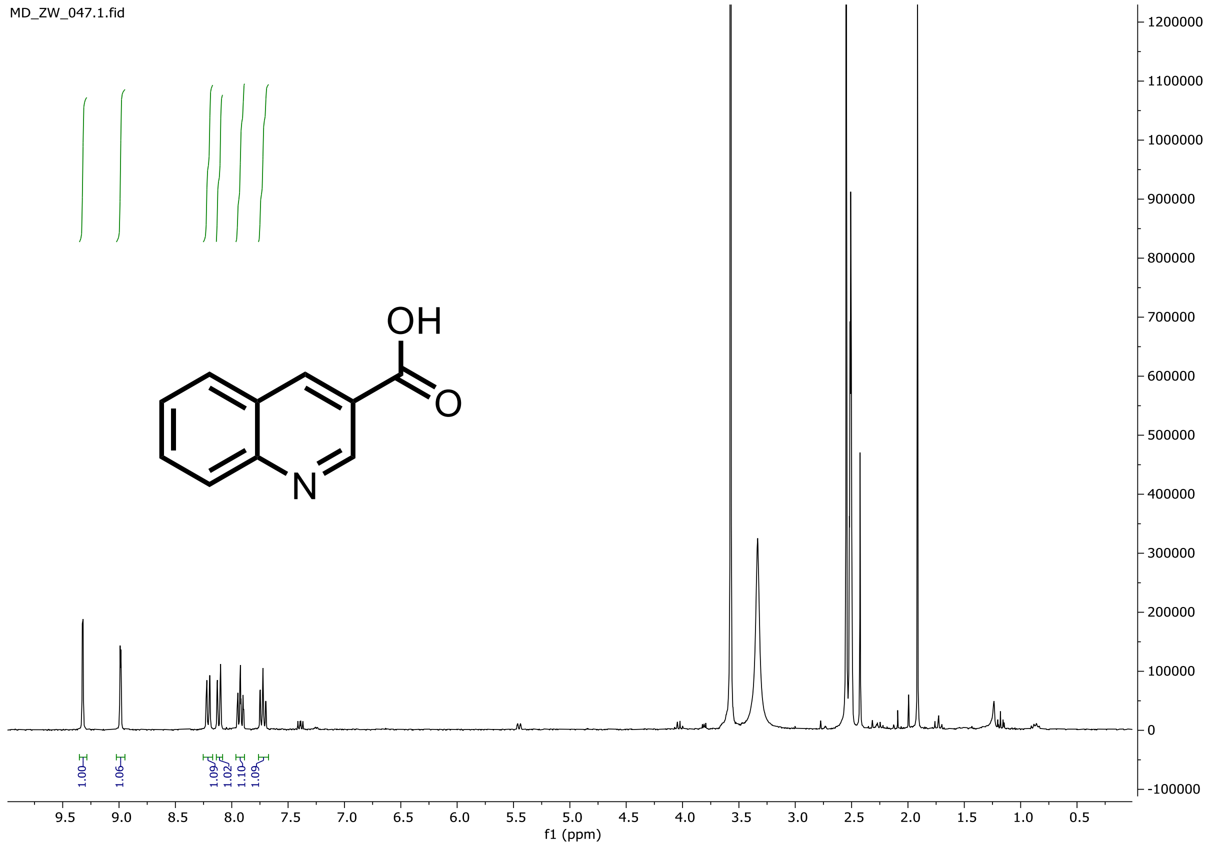
MD_ZW_047.1.fid
OH
O
N
f1 (ppm)

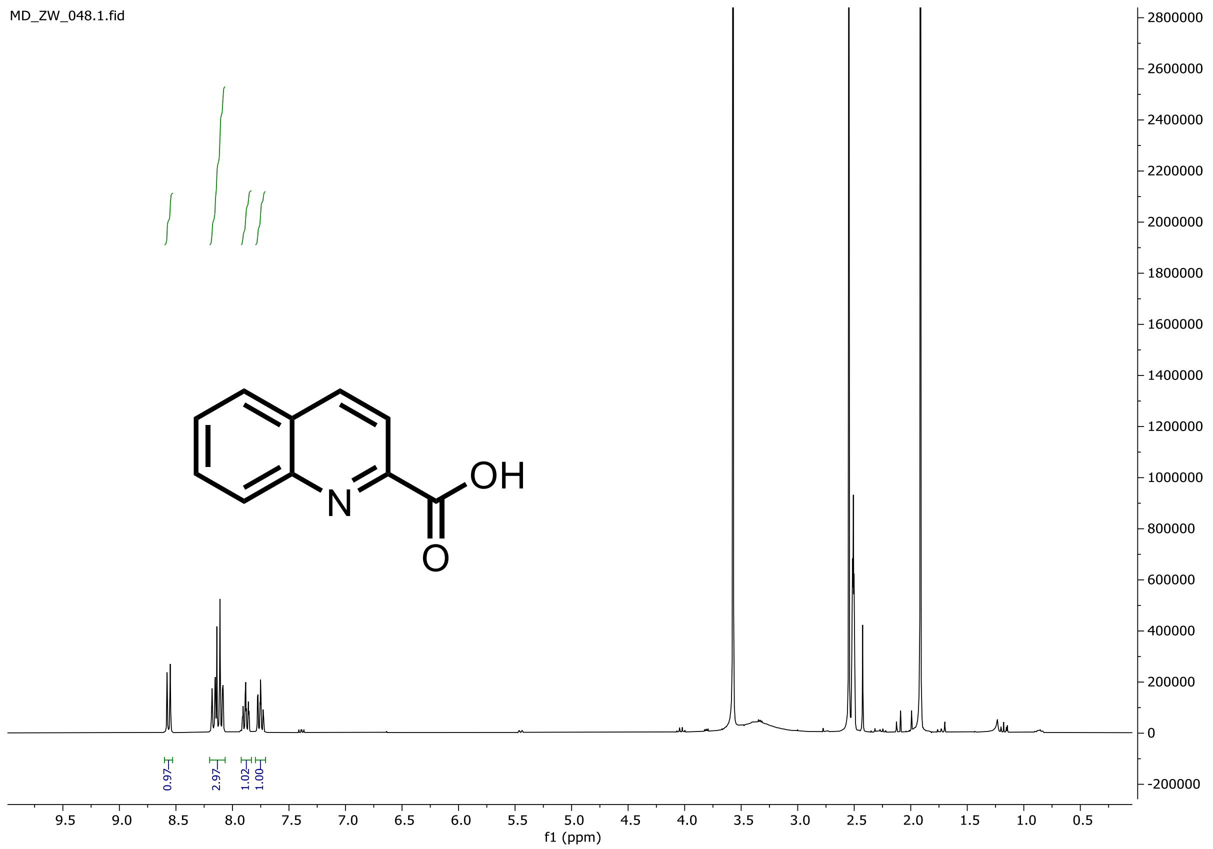
MD_ZW_048.1.fid
OH
N
O
0.97
2.97
1.02
1.00
9.5 9.0 8.5 8.0 7.5 7.0 6.5 6.0 5.5 5.0 4.5 4.0 3.5 3.0 2.5 2.0 1.5 1.0 0.5
f1 (ppm)
2800000
2600000
2400000
2200000
2000000
1800000
1600000
1400000
1200000
1000000
800000
600000
400000
200000
0
-200000

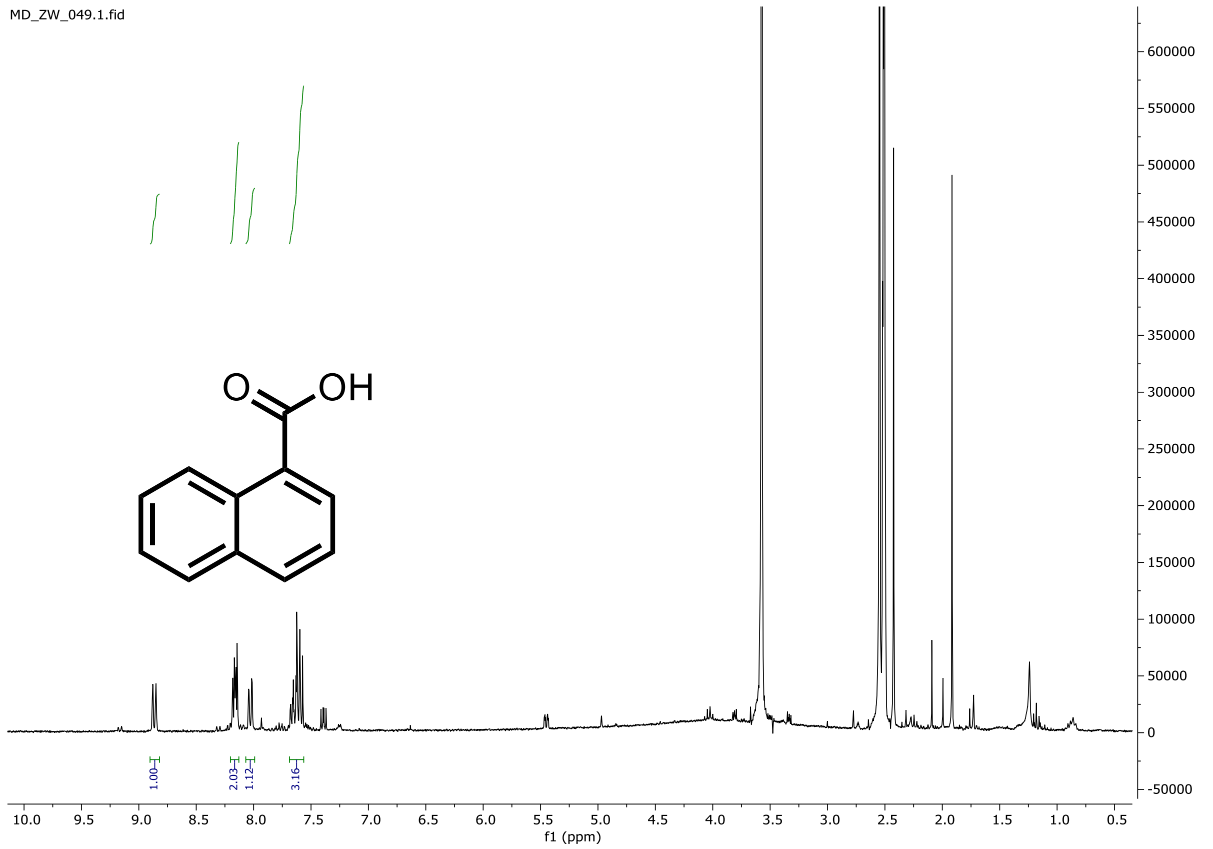
MD_ZW_049.1.fid
O
OH
1.00
2.03
1.12
3.16
10.0 9.5 9.0 8.5 8.0 7.5 7.0 6.5 6.0 5.5 5.0 4.5 4.0 3.5 3.0 2.5 2.0 1.5 1.0 0.5
f1 (ppm)
600000
550000
500000
450000
400000
350000
300000
250000
200000
150000
100000
50000
0
-50000

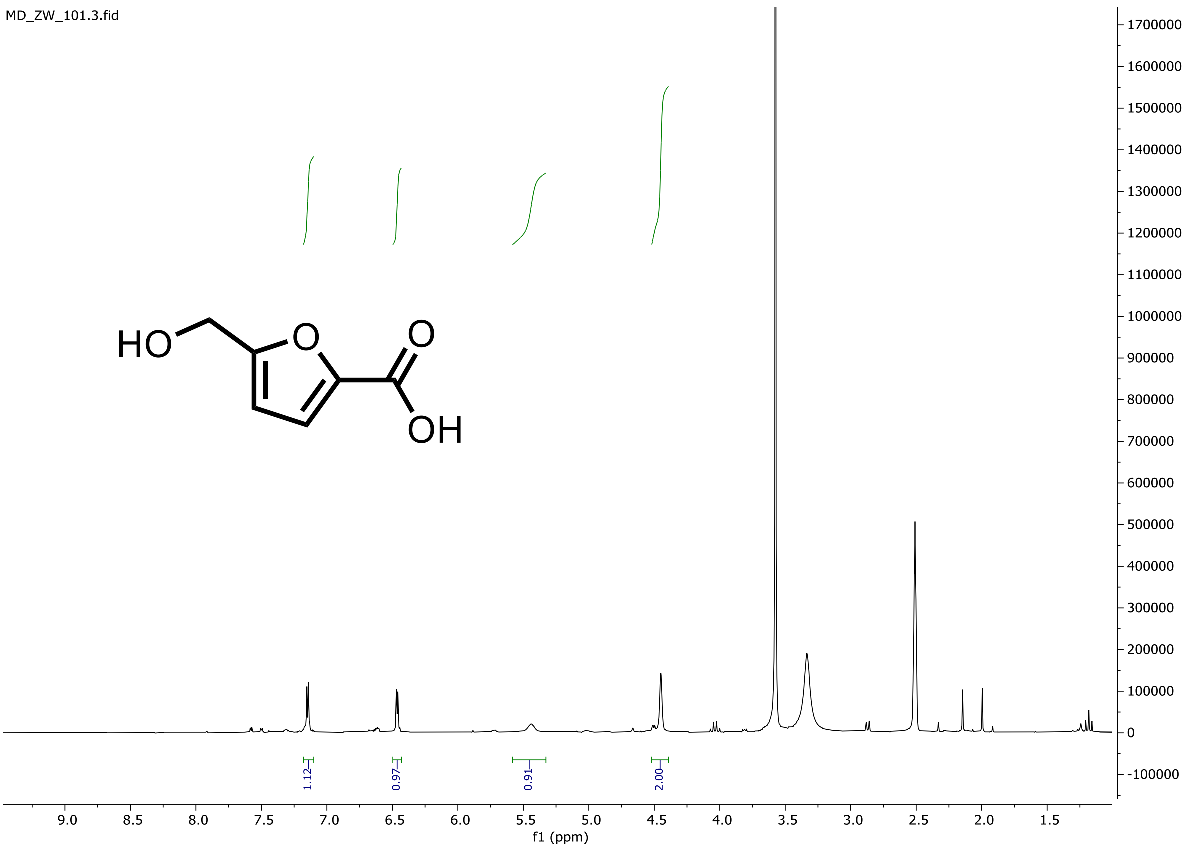
MD_ZW_101.3.fid
HO
O
O
OH
1.12
0.97
0.91
2.00
9.0 8.5 8.0 7.5 7.0 6.5 6.0 5.5 5.0 4.5 4.0 3.5 3.0 2.5 2.0 1.5
f1 (ppm)
1700000 1600000 1500000 1400000 1300000 1200000 1100000 1000000 900000 800000 700000 600000 500000 400000 300000 200000 100000 0 -100000

10.References

(1) Yamamoto, H.; Kudoh, M. Novel chiral tool,(R)-2-octanol dehydrogenase, from Pichia finlandica: purification, gene cloning, and application for optically active α-haloalcohols. *Appl. Microbiol. Biotechnol.* **2013**, *97*, 8087-8096.

(2) Höffken, H. W.; Duong, M.; Friedrich, T.; Breuer, M.; Hauer, B.; Reinhardt, R.; Rabus, R.; Heider, J. Crystal structure and enzyme kinetics of the (S)-specific 1-phenylethanol dehydrogenase of the denitrifying bacterium strain EbN1. *Biochemistry* **2006**, *45*, 82-93.

(3) Lavandera, I.; Kern, A.; Schaffenberger, M.; Gross, J.; Glieder, A.; de Wildeman, S.; Kroutil, W. An exceptionally DMSO-tolerant alcohol dehydrogenase for the stereoselective reduction of ketones. *ChemSusChem* **2008**, *1*, 431-436.

(4) Cannio, R.; Rossi, M.; Bartolucci, S. A few amino acid substitutions are responsible for the higher thermostability of a novel NAD+-dependent bacillar alcohol dehydrogenase. *Eur. J. Biochem.* **1994**, *222*, 345-352.

(5) Matsumoto, J.; Higuchi, M.; Shimada, M.; Yamamoto, Y.; Kamio, Y. Molecular cloning and sequence analysis of the gene encoding the H2O-forming NADH oxidase from Streptococcus mutans. *Biosci. Biotechnol. Biochem.* **1996**, *60*, 39-43.

(6) Duan, Y.; Wu, C.; Chowdhury, S.; Lee, M. C.; Xiong, G.; Zhang, W.; Yang, R.; Cieplak, P.; Luo, R.; Lee, T.; et al. A point-charge force field for molecular mechanics simulations of proteins based on condensed-phase quantum mechanical calculations. *J. Comput. Chem.* **2003**, *24*, 1999-2012.

(7) Birmingham, W. R.; Turner, N. J. A Single Enzyme Oxidative "Cascade" via a Dual-Functional Galactose Oxidase. *ACS Catal.* **2018**, *8*, 4025-4032.

(8) Li, J.; Duran, C.; Pogranyi, B.; Cornish, K. A. S.; Cartwright, J.; Osuna, S.; Unsworth, W. P.; Grogan, G. Divergent Oxidation Reactions of E- and Z-Allylic Primary Alcohols by an Unspecific Peroxygenase. *Angew. Chem. Int. Ed.* **2025**, *64*, e202422241.

(9) Shiloach, J.; Fass, R. Growing E. coli to high cell density—a historical perspective on method development. *Biotechnol. Adv.* **2005**, *23*, 345-357.

(10) Schmidt, L. *Physiologie des Menschen*; 2007. DOI: 10.1007/978-3-540-32910-7.

(11) Piorino, F.; Styczynski, M. P. Complex Dependence of Escherichia coli-based Cell-Free Expression on Sonication Energy During Lysis. *ACS Synth. Biol.* **2023**, *12*, 3131-3136.

(12) Kasper, J. C.; Friess, W. The freezing step in lyophilization: physico-chemical fundamentals, freezing methods and consequences on process performance and quality attributes of biopharmaceuticals. *Eur. J. Pharm. Biopharm.* **2011**, *78*, 248-263.

(13) Nwankwo, C. S.; Okpomor, E. O.; Dibagar, N.; Wodecki, M.; Zwierz, W.; Figiel, A. Recent Developments in the Hybridization of the Freeze-Drying Technique in Food Dehydration: A Review on Chemical and Sensory Qualities. *Foods* **2023**, *12*, 3437.

(14) Liew, F. E.; Nogle, R.; Abdalla, T.; Rasor, B. J.; Canter, C.; Jensen, R. O.; Wang, L.; Strutz, J.; Chirania, P.; De Tissera, S. Carbon-negative production of acetone and isopropanol by gas fermentation at industrial pilot scale. *Nat. Biotechnol.* **2022**, *40*, 335-344.
